# Molecular/Nanomechanical Insights into Electrostimulation‐Inhibited Energy Metabolism Mechanisms and Cytoskeleton Damage of Cancer Cells

**DOI:** 10.1002/advs.202207165

**Published:** 2023-04-07

**Authors:** Guohua Qi, Miaomiao Zhang, Jilin Tang, Yongdong Jin

**Affiliations:** ^1^ State Key Laboratory of Electroanalytical Chemistry Changchun Institute of Applied Chemistry Chinese Academy of Sciences Changchun Jilin 130022 China; ^2^ School of Applied Chemistry and Engineering University of Science and Technology of China Hefei Anhui 230026 China

**Keywords:** atomic force microscopy, cancer cells, electrical stimulation, energy metabolism

## Abstract

Inhibiting energy metabolism of cancer cells is an effective way to treat cancer but remains a great challenge. Herein, electrostimulation (ES) is applied to effectively suppress energy metabolism of cancer cells to induce rapid cell death, and deeply reveal the underlying mechanisms at the molecular and nanomechanical levels by combined use of fluorescence imaging and atomic force microscopy. Cancer cells are found significantly less tolerant to ES than normal cells; and ES causes “domino effect” to induce mitochondrial dysfunction to impede electron transport chain (ETC) and tricarboxylic acid (TCA) cycle pathways, leading to fatal energy‐supply crisis and death of cancer cells. From the perspective of cell mechanics, the Young's modulus decreases and cytoskeleton destruction of MCF‐7 cell membranes caused by F‐actin depolymerization occurs, along with down‐regulation and sporadic distribution of glucose transporter 1 (GLUT1) after ES. Such a double whammy renders ES highly effective and promising for potential clinical cancer treatments.

## Graphical abstract

Electrostimulation (ES) effectively suppresses energy metabolism of cancer cells to induce rapid cell death, the underlying mechanisms of which are revealed at the molecular and nanomechanical levels by combined use of fluorescence imaging and atomic force microscopy. The results show that glucose metabolism in cancer cells are destroyed and the Young's modulus decreases, and cytoskeleton destruction of cell membranes occurs.

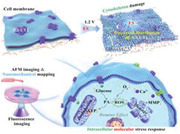

## 1 Introduction

Cellular bioenergetics is closely related to cell fates such as cell proliferation, differentiation, and apoptosis,^[^ ^1^, ^2^, ^3^ ^]^ and the development of metabolic diseases.^[^ ^4^ ^]^ Especially, tumor initiation and progression are accompanied by accelerated nutrient uptake and intensive alterations in cellular energy metabolism.^[^ ^5^ ^]^ Cancer cells accommodate dynamically fuel utilization to satisfy with their requirements of cellular function, proliferation, and survival.^[^ ^6^ ^]^ Consequently, effective inhibition of energy metabolism pathways of cancer cells could be a critical and effective way to fight cancer.

Electrical stimulation (ES) with the superiorities of low damage, easy and precise parameter control, low induction of immune response, and repeatable operation, has been applied to regulate cellular activities in tissue engineering.^[^ ^7^ ^]^ It also has advantages to modulate cell proliferation, migration, cell differentiation, wound healing, and reprogramming for nerve regeneration^[^ ^8^, ^9^, ^10^ ^]^ over other mechanical or chemical approaches. Compared with other commonly used methods to inhibit energy metabolism (photothermal treatment, photodynamic therapy, sonodynamic therapy, chemotherapy, etc.), ES has obvious advantages, such as no need to prepare any nanomaterials, strong controllability, fast response, time‐saving, and fewer side effects. Recently, we found preliminarily that cancerous cells are more susceptible to death than normal cells under certain ES conditions.^[^ ^11^, ^12^, ^13^, ^14^ ^]^ Nevertheless, a deeper understanding of how electric field may affect energy metabolism of cancerous cells and how the ES‐induced energy metabolic program may eventually modulate cytoskeleton dynamics of cells is still lacking and need further investigations.

**Scheme 1:**
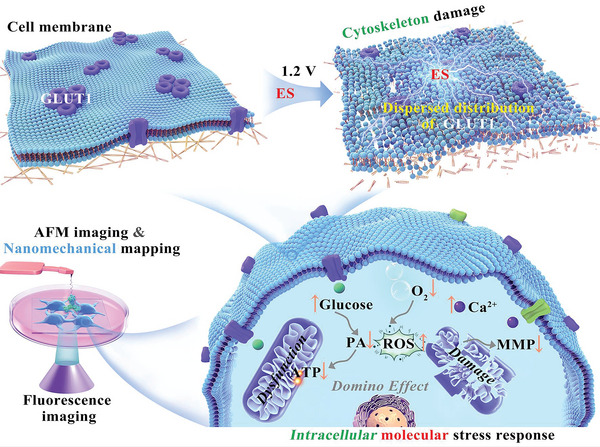
Schematic of molecular and nanomechanical insights into ES‐inhibited energy metabolism mechanisms and cytoskeleton damage of cancer cells, revealed by combined use of fluorescence imaging and atomic force microscopy.

Herein, we report the use of mild ES (at ≈1.2 V) to trigger “domino effect” on mitochondrial dysfunction of cells for killing cancer cells and provide mechanistic insights into the cellular molecular events and nanoscopic variation of cytomembrane and the cytoskeleton of cancer cells under ES process, by combined use of fluorescence imaging and force‐distance curve‐based atomic force microscopy (FD‐based AFM), as depicted in **Scheme** **1**. FD‐based AFM as a powerful nanoscopic platform allows imaging of biological system include cells and biomolecules and simultaneous mapping of their multiple mechanical properties at (sub)nanometer resolution with micro‐/pico‐newton sensitivity.^[^ ^15^, ^16^ ^]^ As AFM tip can be tailor‐made via covalent attachment of biomolecules to get specific functions,^[^ ^17^ ^]^ FD‐based AFM has been intensively used for investigation of ligand‐receptor interactions on diverse systems ranging from biomolecule‐grafted model surfaces to cellular receptors, virus, and bacterial.^[^ ^18^, ^19^, ^20^ ^]^ However, the effect of ES on morphology, mechanical properties, and related energy metabolism of cancer cells have never been studied by the combined use of AFM. The results of this study showed that mechanistically glycolysis and pentose phosphate pathways of glucose metabolism in cancer cells were destroyed after ES under 1.2 V, leading to upregulation of intracellular glucose concentration which is difficult to be converted into bioenergy of Adenosine Triphosphate (ATP) to support tumor cell survival. Attractively, quantitative nanomechanical mapping of FD‐based AFM clearly revealed that the Young's modulus of cancer cells were gradually reduced with increasing voltage, as the blocked production of ATP under high potential which induces cytoskeleton destruction. Furthermore, the expression level of glucose transporter 1 (GLUT1) on cell membrane was reduced and the distribution varied from cluster to dot with increasing voltage as revealed by molecular recognition characterization of AFM.

## 2 Results and Discussion

### 2.1 Domino Effect of ES‐Induced Mitochondrial Dysfunction

**Figure 1:**
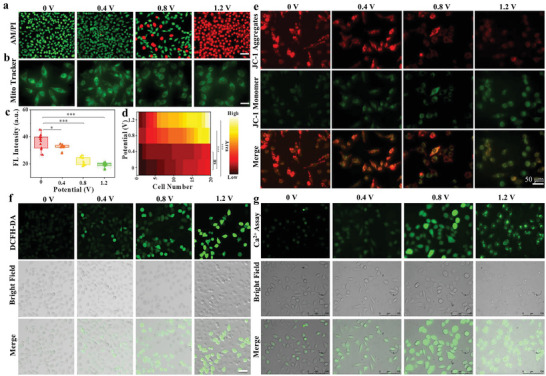
Fluorescence images of MCF‐7 cells stained with a) living/dead dye and b) Mito Tracker after the ES treatment of different voltages for 5 min. c) Perinuclear area of MCF‐7 cells calculated after the ES treatment under different voltages. ***p < 0.0001 represents the statistical significance. d) The fluorescence intensity of Mito Tracker within MCF‐7 cells treated with different voltages for 5 min. *p < 0.05, ***p < 0.0001. *p‐*values were calculated by Graphpad Prism. e) Fluorescence imaging of MMP within MCF‐7 cells using JC‐1 assay kit after the ES treatment with different voltages for 5 min. f) The ROS fluorescence imaging within MCF‐7 cells stained with DCFH‐DA after the ES under different voltages for 5 min. The scale bar is 50 µm. g) The Ca^2+^ fluorescence imaging within MCF‐7 cells stained with Ca^2+^ assay kit after the ES for 5 min under different voltages. The scale bar is 50 µm.

Prior to check the ES effect on mitochondrial functions, we performed viability test of cells during ES process at varied voltages by live/dead cell fluorescence dye staining (calcein‐AM/propidium iodide (PI)). As seen from **Figure** **1**a and Figure S1, Supporting Information, the positive correlation between mortality rate of three tested cell lines (MCF‐7, HepG2, and HeLa cells) and voltages elevation was confirmed by fluorescence imaging. Simultaneously, the cell viability of three tested cell lines also demonstrated that cell viability of three cell lines was gradually reduced with the voltages boosted (Figure S2, Supporting Information). The mitochondria as dynamic organelles grow, divide, and fuse in cells.^[^ ^21^ ^]^ These dynamic processes control the size, shape, and number of mitochondria, which in turn administrate the distribution, function, and turnover of mitochondria. Consequently, the dynamic process of mitochondria was checked using the commercial dye of Mito‐Tracker Green during the ES process (Figure 1b), whose fluorescence intensity was not affected by the mitochondrial membrane potential.^[^ ^22^ ^]^ The fluorescence intensity of Mito‐Tracker Green within mitochondria of MCF‐7 cells was gradually decreased with increasing the stimulation voltage (Figure 1b,c), demonstrating that the mitochondrial numbers of cells were correspondingly reduced. The reticular networks by elongated mitochondria were observed from healthy cells, whereas for cells treated with ES at 0.8 and 1.2 V, almost all of them emerged a shorter length along with cell center area amplified (Figure 1b,d), indicative of somehow damage of mitochondria. Furthermore, the interferential effect of ES on mitochondrial functions was examined under different voltages. As mitochondrial membrane potential (MMP) is an indicator of mitochondrial functions, the MMP in the three tested cell lines (MCF‐7, HepG2, and HeLa cells) treated with different voltages was assessed using the JC‐1 kit (Figure 1e, Figure S3, Supporting Information). All the three cell lines exhibited strong green fluorescence and the red fluorescence intensity relatively weakened after ES treatments from 0 to 1.2 V, demonstrating the depolarization of cellular MMP by ES at 1.2 V. The ratio of the red to green signals in the 1.2 V group from MCF‐7 cells was found ≈6‐fold lower than that of control group (Figure S4, Supporting Information). As MMP damage can trigger reactive oxygen species (ROS) storm in cells,^[^ ^23^ ^]^ the ROS levels in three cell lines after the ES treatments under different voltages (0, 0.4, 0.8, and 1.2 V) were evaluated using the DCFH‐DA assay kit.

As shown in Figure 1f and Figure S5, Supporting Information, the green fluorescence signal of cells gradually increased with the increase of stimulation voltage, manifesting that ES can promote the up‐regulation of ROS level in MCF‐7, HepG2, and HeLa cells. It has been reported that the ROS level elevated can induce the intracellular calcium overload. Remarkably, the fluorescence intensity of calcium ions (Ca^2+^) in mitochondria was found gradually enhanced with increasing voltage and the Ca^2+^ level peaked at 0.8 V (Figure 1g and Figure S6, Supporting Information), while the fluorescence signal of Ca^2+^ in cells was slightly decreased after 1.2 V stimulation due to severe damage of cell membranes. The results confirmed that the ES can trigger a domino effect of mitochondrial damage to lead to functional dysfunction.

### 2.2 Electrical Stimulation Inhibits Energy Metabolism of Cancerous Cells

**Figure 2:**
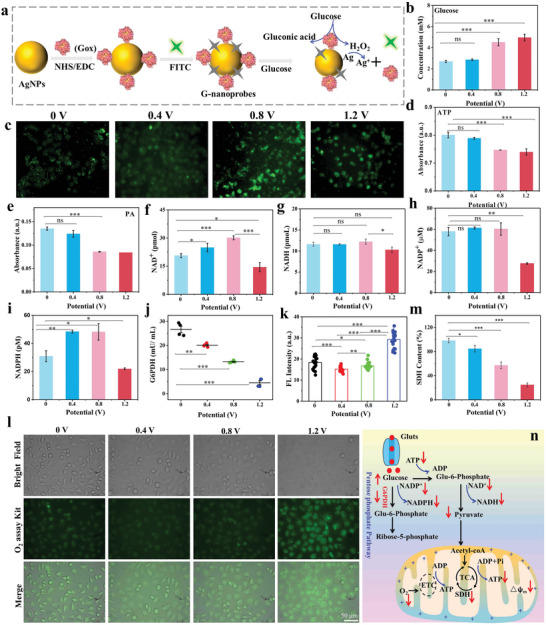
a) Schematic illustration of preparation process of G‐nanoprobes for glucose sensing. b,c) The fluorescence imaging of glucose and concentration variation within MCF‐7 cells detected using G‐nanoprobes under different voltages for 5 min. d–g) The content of d) ATP, e) PA, f) NAD^+^, and g) NADH within MCF‐7 cells treated for 5 min under different voltages. h,i) The content variations of NADP^+^ and NADPH within MCF‐7 cells after the ES under different voltages for 5 min. j) The enzymatic activity variation of G6PDH within MCF‐7 cells during ES under different voltages for 5 min. k,l) Evolution of O_2_ variation within MCF‐7 cells by RDPP probe after ES under different voltages for 5 min. m) The SDH content variation within MCF‐7 cells during ES under different voltages for 5 min. *p < 0.05, **p < 0.001, and ***p < 0.0001 represents the statistical significance. n) Schematic illustration of the proposed mechanisms on the ES‐induced/‐triggered inhibition effect on the cellular energy metabolisms.

Mitochondrial matrix Ca^2+^ plays a key role in energy metabolism of cells.^[^ ^24^, ^25^ ^]^ Consequently, two major energy generation pathways, namely oxidative phosphorylation and glycolysis, were evaluated. First, the concentration of cellular glucose as the obligatory energy substrate was estimated after ES using a glucose nanoprobe (G‐nanoprobe) as designed in **Figure** **2**a. The fluorescence and surface‐enhanced Raman spectroscopy (SERS) dual‐responsive G‐nanoprobe was first prepared and characterized. The designed G‐nanoprobe possessed superior repeatability, biocompatibility and high specificity and sensitivity, which are beneficial for single‐cell glucose detection (Figures S7–S9, Supporting Information). The optimal incubation time of G‐nanoprobes with three tested cell lines (H8, HeLa and MCF‐7 cells) in glucose‐free medium was selected as 4 h (Figures S10 and S11, Supporting Information). The high cell viability of cell lines cultured with glucose‐free medium with or without G‐nanoprobes was discovered, while applied external electric field can selectively kill cancer cells at 1.2 V (Figure S12, Supporting Information). The G‐nanoprobes entered into the cells were then used to assess the intracellular glucose levels during ES process. The SERS intensity at 1323 cm^−1^ from G‐nanoprobes in MCF‐7 cells was gradually decreased with ES voltage amplified (Figure S13, Supporting Information), and quantitative analysis of glucose concentrations within cells showed that the glucose level was remarkably increased from ≈2.7 ±0.1 mm of untreated cells to ≈4.5±0.34 and 4.96 ± 0.32 mm, after the ES at 0.8 and 1.2 V for 5 min (Figure 2b), respectively. The fluorescence signals of glucose were also gradually enhanced within cells (Figure 2c). And notably, the intracellular glucose concentrations of MCF‐7 cells were apparently elevated with ES time prolonged at 1.2 V (Figure S14, Supporting Information). Clearly, we found that the glucose level within cancerous cells was obviously higher than that in normal cells after the ES at 1.2 V for 5 min (Figures S15 and S16, Supporting Information). Moreover, the ATP level within MCF‐7 cells was gradually decreased with the increase of ES voltage (Figure 2d). Therefore, we assume that the applied external electric field can restrain glucose metabolism within cancer cells to induce energy requirements obstacle, which is fatal to cancer cell survival.

To test the hypothesis, we first explored the aerobic glycolysis pathway, which was termed as Warburg effect, during the ES process. Warburg effect is typical of glucose metabolism in cancer cells, in which glucose is mainly processed into lactate.^[^ ^26^, ^27^ ^]^ Specifically, the contents of pyruvic acid (PA) within MCF‐7 cell were measured using the PA assay kit during ES process under different voltages, which clearly indicated that the conversion capability of glucose to pyruvate was obviously weakened for MCF‐7 cells after the ES at 1.2 V (Figure 2e). Meanwhile, the expression of nicotinamide adenine dinucleotide (oxidized, NAD^+^; reduced, NADH) was also evaluated in the glycolysis route. It is a significant pyridine nucleotide, which plays an extremely important role in the energy production through redox reaction. The down‐regulation of NAD^+^ and NADH within MCF‐7 cells after ES at 1.2 V was observed, relative to cells treated at 0 V (Figure 2f,g). Furthermore, the NADH/NAD^+^ ratio, which serves as a pivotal indicator and reflects the overall redox state of energy metabolism,^[^ ^28^ ^]^ was calculated before and after the ES at 1.2 V for 5 min (Figure S17a, Supporting Information). The analysis revealed a significant increase of the intracellular NADH/NAD^+^ ratio from 0.56 to 0.72 after ES at 1.2 V, suggesting that the intracellular redox balance was destroyed. It further restrained transferring energy in nutrients to the electron transport chain (ETC) to produce sufficient electrochemical gradient that drives ATP synthesis. Subsequently, alternative glucose metabolic pathways, pentose phosphate pathways (PPP), were further investigated in this study. Nicotinamide adenine dinucleotide phosphate (NADP) as a ubiquitous coenzyme was involved in numerous redox systems existing in either oxidized (NADP^+^) or reduced (NADPH) states formed in PPP.^[^ ^29^ ^]^ We observed an obvious decrease in the expression of NADPH and NADP^+^ in MCF‐7 cells after ES at 1.2 V (Figure 2h,i). Simultaneously, the NADPH/NADP^+^ ratio calculated was ≈0.53 at 0 V and ≈0.8 at 1.2 V (Figure S17b, Supporting Information), respectively, demonstrating the disruption of redox homeostasis due to the elevation of cellular ROS level. Since glucose‐6‐phosphate dehydrogenase (G6PD) is an important protective enzyme, which is responsible for retaining adequate levels of the major cellular reducing agent NADPH, the enzyme activity of G6PD was also examined (Figure 2j), the result of which showed that the enzyme activity of G6PD was gradually inhibited with increasing voltage. The above results showed that the intracellular glucose metabolism abnormity suppressed the ATP energy generation of MCF‐7 cells under the ES at 1.2 V.

Given that NADH and NADPH participate in electron transferring to the downstream proteins and incidentally donate the electrons to the oxygen in the cellular environment, it will lead to the content change of intracellular oxygen (O_2_). Therefore, intracellular O_2_ concentration variations were measured using [Ru(dpp)_3_]Cl_2_ (RDPP) as O_2_ probe after the ES at different voltages, whose fluorescence intensity will be weakened after the interaction with oxygen.^[^ ^30^ ^]^ The fluorescence intensity of RDPP within MCF‐7 cells after the ES at 1.2 V was the strongest in all experimental groups (Figure 2l,k), indicating that intracellular oxygen was significantly reduced relative to other treatments. It strongly affirmed that the ES can effectively suppress the aerobic respiration to block electron transport chain of cancer cells. Additionally, the activity of succinate dehydrogenase (SDH), a mitochondrial enzyme with the unique property to participate in both the TCA cycle and the ETC, was also assessed using MTT assay (Figure 2m). The results displayed that the activity of SDH decreased obviously with increasing the voltage. Based on all above findings, we proposed a plausible mechanism by which ES inhibits energy metabolism of cancer cells, as depicted in Figure 2n, in which the internalized glucose molecules were first phosphorylated to produce glucose 6‐phosphate participating in the aerobic glycolysis and PPP process; under the ES, glucose molecules are difficult to convert energy due to destruction of redox homeostasis, causing an energy crisis in cancer cells. In the process, mitochondrial ETC and TCA cycle pathways of cells were impeded, which seriously affects the ATP synthesis of the cancerous cells.

### 2.3 Cell Mechanical Properties Measurement by AFM during ES

**Figure 3:**
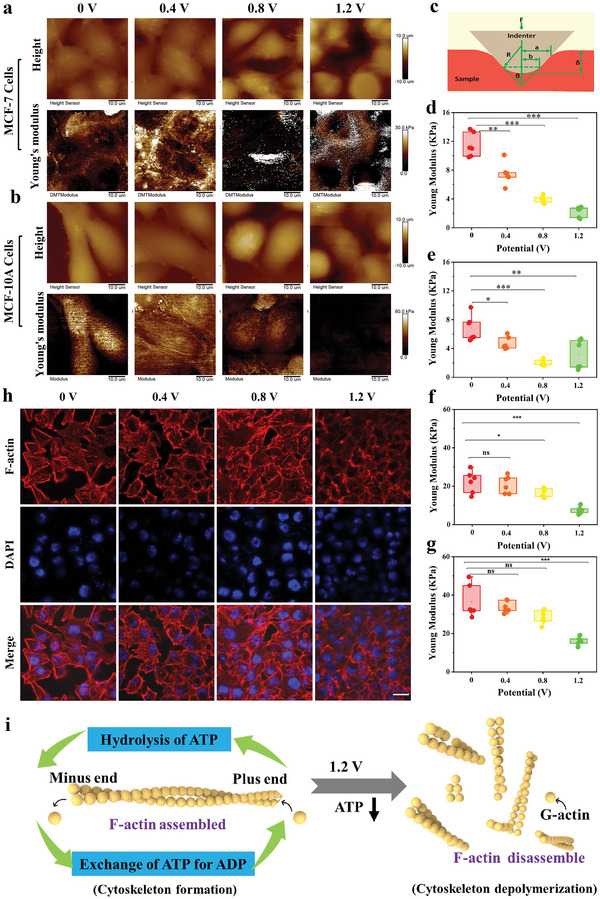
Representative AFM topography images and corresponding Young's modulus images of a) MCF‐7 and b) MCF‐10A cells treated with different voltages from 0 to 1.2 V. c) Geometric definition of a rigid cone with spherical tip. d,e) The statistical analysis of perinuclear and central nuclear areas Young's modulus (fitting with Cone Sphere model) from MCF‐7 cells treated with different voltages for 5 min. f,g) The statistical analysis of perinuclear and central nuclear areas Young's modulus from MCF‐10A cells treated with different voltages (0, 0.4, 0.8, and 1.2 V) for 5 min. Values are expressed as means ± SD. *p < 0.05, **p < 0.001, and ***p < 0.0001 represents the statistical significance. h) The fluorescence imaging of MCF‐7 cells stimulated with different voltages for 5 min. F‐actin (Cy3, red); cell nucleus (DAPI, blue). The scale bar is 20 µm. i) The schematic illustration of the proposed relationship between F‐actin formation and ATP generation.

To explore whether the inhibition of energy metabolism after ES has an impact on the cell mechanical properties, the FD‐based AFM was used to monitor the dynamic mechanical property variation of multiple cell lines under ES at different voltages. Young's modulus is a crucial mechanical property of mammalian cells, which reflects the stiffness and elasticity of cells and is closely related to the physiological state of cells. First, we performed AFM imaging for morphological and mechanical characterizations of two cancerous cell lines (MCF‐7, HeLa cells) and two normal cell lines (MCF‐10A, H8 cells) after ES at different voltages (**Figure** **3**a,b and Figure S18a, Supporting Information). From the morphology mappings of MCF‐7, MCF‐10A, HeLa, and H8 cells (Figure 3a,b and Figure S18a, Supporting Information), we observed that the pseudopods of three cell lines were gradually reduced and the height of cells was increased with increasing voltage. Additionally, surface wrinkles and secretions of MCF‐7 cells were more evidently increased after ES at 1.2 V compared with the other two different normal cells. Importantly, the mechanical properties of four tested cell lines emerged remarkable transformation in the corresponding Young's modulus mappings, demonstrating that the Young's modulus values of MCF‐7, HeLa, MCF‐10A, and H8 cells were observably decreased with the increase of ES voltage. The Young's modulus values of cells were calculated based on force–distance curves by fitting the cone sphere model (Figure 3c). As seen from the statistical results of perinuclear areas of MCF‐7 cells, the Young's modulus values gradually decreased from 11.5 ± 1.7 to 2.2 ± 0.7 KPa with voltage increased from 0 to 1.2 V (Figure 3d), with a decrease of nearly 81%. Notably, the Young's modulus in central nuclear areas of MCF‐7 cells were gradually reduced from 6.9 ± 1.8 (at 0 V) to 2.0 ± 0.4 KPa (at 0.8 V), with a reduction of ≈71%, while it slightly raised to 3.2 ± 2.0 KPa at a high voltage of 1.2 V that might be due to the shrinkage of the cell nucleus at high voltage (Figure 3e). The Young's modulus values of perinuclear areas from HeLa cells was reduced by 88.5% after ES at 1.2 V, compared with control group (Figure S18b, Supporting Information). Similarly, the Young's modulus in central nuclear areas of HeLa cells after ES under different voltages were also calculated from 69.2 ± 9.2 (at 0 V) to 19.4 ± 11.54 KPa (at 0.8 V), with decreasing of 72% (Figure S18c, Supporting Information). Shrinkage of the cell nucleus from HeLa cells at high voltage (at 1.2 V) is consistent with MCF‐7 cells due to slight increase of Young's modulus in central nuclear areas. Meanwhile, Young's modulus values in perinuclear and central nuclear areas of MCF‐10Awere also calculated, the result of which indicates that their Young's modulus values were also gradually reduced with ES voltage increasing from 0 to 1.2 V (Figure 3f,g). The decrease of Young's modulus value for MCF‐10A cells after ES at 1.2 V is nearly 66% (from 22.22 ± 5.73 to 7.55 ± 1.80 KPa) in perinuclear regions, and is about 56% (from 36.55 ± 8.50 to 15.98 ± 2.08 KPa) in central nuclear areas, compared with control groups. Simultaneously, the Young's modulus values of perinuclear and central nuclear areas for H8 cells after ES at different voltages were also calculated (Figure S18d,e, Supporting Information). In perinuclear areas, the Young's modulus values of H8 cells gradually decreased nearly 55% with voltage increased from 0 to 1.2 V (Figure S18d, Supporting Information), and in central nuclear areas is nearly 52% (Figure S18e, Supporting Information). All these results implied that the cancer cells are more sensitive to ES than normal cells from a cellular mechanics perspective.

Cytoskeleton is a fiber‐like scaffold structure throughout the whole cell, which ensures the cell structural and functional integrity and contributes to mechanical properties of cells.^[^ ^31^ ^]^ F‐actin network as the main components of the cytoskeleton near the membrane participates in the responses to stimulation in cell surroundings by dynamical rearrangement, which changes cell mechanical properties indirectly. Consequently, the F‐actin state of MCF‐7 cells after ES at different voltages was further checked using fluorescence‐labeled imaging (Figure 3 h). The results showed that the F‐actin levels of cells were significantly lowered with increasing voltage from 0 to 1.2 V, demonstrating that the cytoskeleton has been rearranged or even depolymerized after the ES. Normally, F‐actin is assembled by polymerization of monomeric G‐actin with bound ATP, on which the hydrolysis reaction occurs to cleave ATP resulting in a highly stable filament, followed by the release of inorganic phosphate (Pi), giving rise to the disassembly of F‐actin (Figure 3i).^[^ ^32^, ^33^ ^]^ Clearly, the ES could perturb the energy metabolism within MCF‐7 cells and induce the decline of ATP levels, and further causes a decrease in the cellular Young's modulus through further disruption of the cytoskeletal structure of MCF‐7 cancer cells.

### 2.4 Molecular Recognition Imaging of GLUT1 and Glucose Uptake Capacity of MCF‐7 Cells during ES

**Figure 4:**
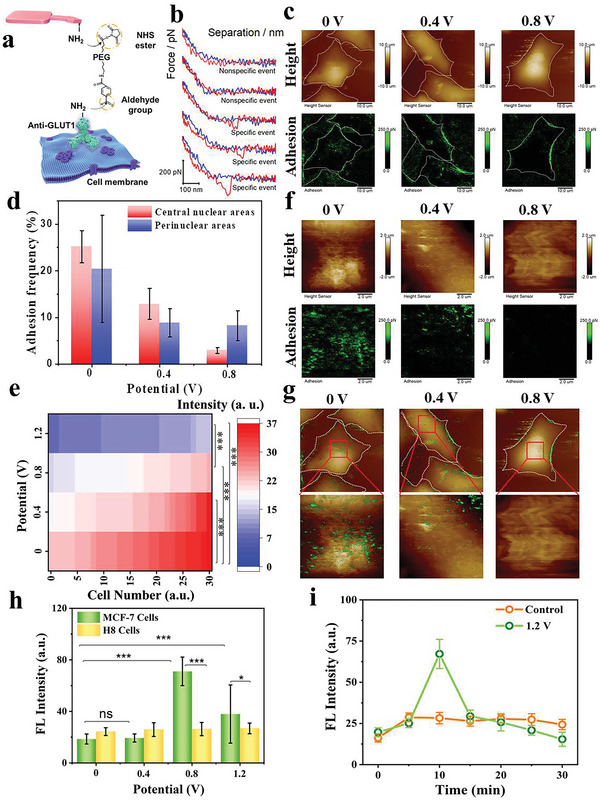
a) Schematic illustration of molecular recognition imaging strategy of GLUR1 on MCF‐7 cell surface. b) Representative force curves of the nonspecific and specific events. c) Morphology (height) and corresponding recognition image (Adhesion) of GLUT1 on the surface of MCF‐7 cells after the ES treatment under different voltages for 5 mm (size: 50 µm × 50 µm). d) Statistics of GLUT1 recognition probability of MCF‐7 cells in nuclear and perinuclear regions after the ES treatment with different voltage for 5 min. e) The fluorescence intensity heat maps of GLUT1 on cell membrane from MCF‐7 cells treated with ES at different voltages for 5 min. f) Local amplified morphology (Height) and corresponding GLUT1 recognition image (Adhesion) of the nuclear regions of MCF‐7 cells surface after the ES under different voltages for 5 min (size: 10 µm × 10 µm). g) Merged images of GLUT1 recognition and morphology of MCF‐7 cells under different voltage treatment for 5 min. h) The fluorescence intensity of 2‐NBDG within MCF‐7 and H8 cells after the ES under different voltages for 5 min. Values are expressed as means ± SD. *p < 0.05 and ***p < 0.001 represents the statistical significance. i) The fluorescence intensity of 2‐NBDG within MCF‐7 cells incubated with glucose‐free DMEM before and after the 1.2 V ES for different incubation time.

To deeply gain an insight into regulatory mechanisms of energy metabolism associated with the ES process, the distribution and expression of specific glucose transporter1 (GLUT1), a protein machine for transporting glucose into cells, were investigated by AFM‐based molecular recognition imaging and fluorescence imaging. In this study, we functionalized the AFM tips with anti‐GLUT1 monoclonal antibody using the heterobifunctional PEG linker (**Figure** **4**a). During molecular recognition imaging, the antibody on the AFM tip will interact with the GLUT1 on the cell surface to form a complex when they contact with each other. When the probe leaves cell surface, the complex dissociates, and the dissociative force will be recorded in the force–distance curve and reflected in the corresponding position in the adhesion map. Thus, GLUT1 on the cell surface can be recognized at single‐molecule level via its specific adhesion interactions with antibody on AFM tip, and visually localized in the adhesion map (Figure 4b). Since the cell membrane state of MCF‐7 cells became very poor after the ES at 1.2 V, which was unfavorable for the recognition imaging with specific adhesion as a signal, the GLUT1 recognition imaging of MCF‐7 cells was only performed under voltages of 0, 0.4, and 0.8 V. Figure 4c shows the AFM topography images and corresponding adhesion maps of MCF‐7 cells under different voltages, revealing that GLUT1 expression decreased which was reflected by a decreased adhesion probability with the increase of voltage. Simultaneously, the adhesion probabilities in nuclear and the perinuclear regions were calculated respectively (Figure 4d). The adhesion probability of the functionalized probes in the nuclear regions of MCF‐7 cells was decreased nearly 88%, from 25.2 ± 3.4% to 2.9 ± 0.6% with the voltage increasing from 0 to 0.8 V. The adhesion probabilities in perinuclear regions of MCF‐7 cells were decreased from 20.4 ± 11.5% to 8.2 ± 3.2%. The expression levels of GLUT1 on MCF‐7 cells were further tested using the immunofluorescence imaging (Figure S19, Supporting Information). Similarly, the GLUT1 expression levels were obviously reduced with increasing voltage from 0 to 1.2 V. The fluorescence intensity analysis was consistent with adhesion probability analysis, confirming that the applied electric field can decrease the GLUT1 expression of MCF‐7 cancer cells (Figure 4e); whereas we found that the GLUT1 expression levels of H8 normal cells basically remained unchanged after the ES under different voltages, with only slight increase of GLUT1 level at 0.8 V (Figures S20 and 21, Supporting Information).

To further reveal GLUT1 distribution pattern on cell membranes after the ES at different voltages, we investigated the distribution of GLUT1 on MCF‐7 cells at single molecule level by molecular recognition imaging at a much higher resolution. The distribution status of GLUT1 on the selected nuclear region of MCF‐7 cell surface were estimated after the ES treatment, which demonstrated that the clustering distribution of GLUT1 on cell membrane was gradually transformed into sporadic distribution with voltages elevated from 0 to 0.8 V (Figure 4f). The merged images of AFM topography and adhesion maps of MCF‐7 cells confirmed more intuitively this conclusion (Figure 4g). It might be related to the F‐actin disruption caused by ES, since the distribution of GLUT1 on cell surface could be changed from clusters to dispersed states when the actin cytoskeleton depolymerized by cytochalasin B.^[^ ^34^ ^]^ We further examined the glucose uptake capacity of cells after the ES using fluorescence glucalogue (2‐NBDG), which has the same transporter as glucose but would be phosphorylated to 2‐NBDG‐6‐phosphate trapped in vivo.^[^ ^35^ ^]^ In the test, the fluorescence intensity of 2‐NBDG within cells could reflect glucose uptake capacity of cells. After the ES treatment at 0.8 V, the fluorescence intensity of 2‐NBDG within MCF‐7 cells was evidently increased (Figure S22, Supporting Information and Figure 4h), which might be due to the fact that the sporadic distribution of GLUT1 could facilitate transport of glucose into cells.^[^ ^34^ ^]^ Nevertheless, when the ES voltage was increased to 1.2 V, the membrane of MCF‐7 cells was severely damaged or even broken, resulting in decreased content of intracellular glucose. Simultaneously, the uptake capacity of glucose for MCF‐7 cells was estimated from 2‐NBDG fluorescence imaging after ES treatment at 1.2 V under different incubation time. As shown in Figure 4i, the uptake capacity of glucose for MCF‐7 cells was reached the highest level after ES for incubating time of 10 min, and then gradually decreased. Nevertheless, the uptake capacity of glucose for MCF‐7 cells without ES treatment basically remained the same.

## 3 Conclusion

Here, we proposed a simple and controlled ES means for inhibiting energy metabolism to induce cancer cell death, and deeply revealed underlying mechanisms of inhibited energy metabolism of cancer cells under electrical stimulation by combined use of fluorescence molecular imaging and nanomechanical AFM mapping. The ES can fleetly and effectively cause “domino effect” to trigger ROS storm and Ca^2+^ over loading, which induce fast and effective mitochondrial dysfunction. In this process, redox homeostasis of mitochondria was disturbed to impede ETC and TCA cycle pathways which seriously affects glucose metabolism to further restrain ATP synthesis, leading to a fatal blow to cancer cells. Meanwhile, the cellular cytoskeletal structure of cancer cells was clearly collapsed after the ES at 1.2 V leading to mechanical properties reduction of cells. This is because the decreased energy level of cells after the ES will cause F‐actin depolymerized or disassembled to finally induce the decrease of Young's modulus. And interestingly, the GLUT1 level on cell membrane of MCF‐7 cell were found reduced after the ES and the distribution form of GLUT1 was converted from clusters to sporadic distribution (at 0.8 V), which may contribute to the uptake of glucose by cancer cells to resist the fatal beating of ES feebly. The double whammy of ES on the energy metabolism pathway and cytoskeleton of cancer cells makes the ES a very effective and promising way for cancer treatments. The results and findings provide deep and fundamental insights into the mechanisms of energy metabolism and cell mechanical modulation in cancer cells during ES treatment, and will promote wide applications of the ES approach for potential cancer treatment.

## 4 Experimental Section

### Preparation of Glucose Nanoprobes (G‐Nanoprobes)

First, 10 mm of FITC solution (100 µL) was added into 20 mL of AgNPs to mix and stir for 4 h. Subsequently, the mixed solution was centrifugated at 5500 rpm for 10 min and washed using water by three times to remove unconjugated FITC. The FITC was modified on the surface of AgNPs through Ag—S bonds. After that, the deposition was dispersed into 20 mL water and then 20 µL of EDC (25 mm) and 20 µL of NHS (25 mL) were added into the FITC‐AgNPs solution to stir for 2 h. The mixed solution was centrifugated at 5500 rpm for 10 min. Subsequently, the 40 mg of GO*_x_* was added into the solution (20 mL) to stir for 12 h. Finally, the mixed solution was centrifugated at 5500 rpm for 10 min by three times.

### Ca^2+^ Fluorescence Imaging within MCF‐7 Cells during ES Process

Briefly, 10 µL of Ca^2+^ fluorescent reagent (1 µg µL^−1^) was added into 1.2 mL of DMEM to co‐culture with MCF‐7 cells for 30 min after the ES under different voltages (0, 0.4, 0.8, and 1.2 V) for 5 min. Then, the cells were clearly washed using PBS three times and the cells were observed using the fluorescence microscope under 40× objective.

### Detection of NAD^+^/NADH within MCF‐7 Cells after ES

The contents of NAD+ and NADH within cells were tested using the commercialized assay kits. First, the cells were planted on the ITO glass and then treated with ES under different voltages (0, 0.4, 0.8, and 1.2 V) for 5 min. After that, the cells were rinsed using PBS three times and then lysed by NAD+/NADH extraction solution at room temperature. Subsequently, the solution was centrifugated for 10 min at 12 000 × *g* at 4 °C and the supernatant was used as a sample for test. 20 µL of samples under different conditions were added into 96‐well plate and 20 µL of NAD+/NADH extraction solution were set as control groups. Subsequently, 90 µL of ethanol dehydrogenase working solution was added into each well to incubate in dark for 10 min. Then 10 µL of coloration solution was added into each well to incubate in dark for 30 min. Finally, the absorption values at 450 nm were detected and the concentrations of NAD+ were calculated based on the standard curve.

The NADH measurement was performed according to the following procedure. First, 50–100 µL of samples in a centrifuge tube was heated on a 60 °C‐water bath or PCR instrument for 30 min to decompose the NAD^+^. If insoluble material was existed after heating, the solution was centrifuged at room temperature for 5 min. Then 20 µL of supernatant was used as a sample for detection according to the above procedure.

### Detection of NADP^+^/NADPH within MCF‐7 Cells after ES

Similarly, the intracellular contents of NADP^+^ and NADPH within cells were also detected by the commercialized assay kit. The detection was performed after the cells subjected to electrical stimulation treatment according to the kit instructions. Total NADP and NADPH concentrations were calculated separately from the standard curve, and the NADPH/NADP^+^ ratio was calculated.

### Detection of G6PDH within MCF‐7 Cells after ES

The G6PDH detection was performed by using the commercialized assay kit. Typically, the cells on the ITO glass were washed using the PBS three times and then treated using the G6PDH lysate. After that the mixed solution was centrifugated at 12 000 rpm at 4 °C and the supernatant was collected and used as samples. 50 µL of samples under different conditions were added into each well of 96‐well plate and then 50 µL of work solution of G6PDH were mixed with the samples in each well to incubate for 10 min at 37 °C. The work solution of G6PDH without G6PDH was set as control group. After that the absorption value at 450 nm of mixed solution was measured. The concentrations of G6PDH in samples were calculated based on the obtained standard curve.

### AFM Nanomechanical Measurements of MCF‐7 Cells during ES under Different Voltages

The morphology and nanomechanical properties of cells before and after electrical stimulation were characterized using the Peak Force QNM mode (Nanoscope v9.3) of BioScope Resolve AFM (Bruker, USA). The cells‐seeded ITO was mounted in an AFM sample cell and imaged in serum‐free medium. The probe used for imaging was PFQNM‐LC‐CAL (Bruker, USA), the tip radius was 70 nm, the opening angle was 15°, and the spring constant was calibrated by the manufacturer. During the AFM measurements, the peak force was set to 300 pN, the peak force frequency was 1 kHz, the peak force amplitude was set as 300 nm, the scan rate was 0.3 Hz, and the pixel was 256 × 256.

In this work, the AFM tip can be modeled as a rigid cone with a spherical tip, so the Young's modulus *E* of the cell can be fitted to the force curve using a cone sphere model. Figure 3e displays the geometric definition of such a rigid cone with a spherical tip, where *F* is the force, *R* is the radius of the probe tip, *δ* is the indentation depth, *a* is the radius of contact, and *b* is the spherical contact radius. In addition, the hysteresis effect caused by the hydrodynamic damping of the liquid medium needs to be excluded from the force curve of the raw data by subtracting the background before fitting. The baseline of the force curve was calibrated using the baseline segment at 10–50%, and the fit boundary of the force curve was set to 30–90%.

When *a* > *b*, the Cone Sphere model is described by the following formula

(1) $$\begin{array}{ccc} F & = & 2\frac{E}{\left(1-\upsilon^{2}\right)}\left[a\delta -\frac{a^{2}}{2\tan\theta }\left[\frac{\pi }{2}-a\sin\left(\frac{b}{a}\right)\right]\right.\\ & & \left.-\;\frac{a^{3}}{3R}+{\left(a^{2}-b^{2}\right)}^{1/2}\left(\frac{b}{2\tan\theta }+\frac{a^{2}-b^{2}}{3R}\right)\right] \end{array}$$

where *υ* is the Poisson's ratio (usually 0.2–0.5, 0.5 was used in this experiment). The contact radius *a* and spherical contact radius *b* are derived from the following equations

(2) $$\delta +\frac{a}{R}\left({\left(a^{2}-b^{2}\right)}^{\frac{1}{2}}-a\right)-\frac{a}{\tan\theta }\;\left[\frac{\pi }{2}-a\sin\left(\frac{b}{a}\right)\right]=0$$

(3) b=Rcosθ

### Molecular Recognition Imaging of the Cell‐Surface GLUT1 Protein Using AFM

In this work, GLUT1 proteins on cell surface were recognized by the quantitative nanomechanical imaging at PeakForce QNM mode of BioScope Resolve AFM (Bruker, USA) with the PFQNM‐LC‐CAL probes (Bruker, USA), tips of which were modified with GLUT1 monoclonal antibody. To functionalize the probes, first, they were cleaned in a UV–ozone cleaner for 30 min, rinsed three times with ultrapure water and ethanol in sequence, and dried gently in a stream of argon. Then, the probes were placed in a desiccator, and 50 µL of APTES and 15 µL of TEA (as a catalyst) were used for 2 h vapor deposition and 2 days of curing reaction to introduce amino groups on the probe surface. The probes were then washed three times with chloroform, dried with nitrogen, and immersed in 500 µL of chloroform containing 1 mg of NHS‐PEG_18_‐Acetal crosslinker and 10 µL of TEA for 2 h. Next, the probes were rinsed three times with chloroform, immersed in 10 mg mL^−1^ citric acid for 10 min after drying, and rinsed three times with Milli‐Q water. Then, the tips of probes were placed facing each other in a star shape on parafilm and immersed in 100 µL of 0.1 mg mL^−1^ anti‐GLUT1 monoclonal antibody solution (with 2 µL of freshly prepared NaCNBH_3_ solution, ≈6 wt% NaCNBH_3_ in 10 mm NaOH (aq)) for 1 h at room temperature. Finally, 5 µL of 1 m ethanolamine (pH = 8) was added to the drop and incubated for 10 min to terminate the reaction. After washing three times, the probes were stored in PBS at 4 °C until use. When imaging, the peak force setpoint was 300 pN, the peak force frequency was 0.25 kHz, the peak force amplitude was 300 nm, the scan rate was 0.125 Hz, and the pixels were 256 × 256. Images and force curves were analyzed with NanoScope Analysis v1.9 software (Bruker, USA).

### Glucose Uptake Capability Assessment of Cells after ES under Different Voltages

To verify the glucose uptake capability of the MCF‐7 cells after electrical stimulation, the glucalogue of 2‐NBDG was used in this work, which can accumulate within cells and not be metabolized. Therefore, the fluorescence intensity can indirectly reflect the capacity of the cells to uptake glucose. First, the cells were seeded on the ITO glasses and incubated for 12 h. Subsequently, the cells were treated with voltages from 0 to 1.2 V for 5 min. The cells were washed and co‐cultured with DMEM (without glucose) and 2‐NBDG (200 µm) for 30 min at 37 °C in a humidified atmosphere containing 5% CO_2_. Finally, the cells were cleaned using the PBS three times and observed with fluorescence microscope under excitation wavelength at 510–540 nm.

## Conflict of Interest

The authors declare no conflict of interest.

## Supporting information

Supporting Information

## Data Availability

The data that support the findings of this study are available from the corresponding author upon reasonable request.
