# Supplementary material for: Molecular/Nanomechanical Insights into Electrostimulation‐Inhibited Energy Metabolism Mechanisms and Cytoskeleton Damage of Cancer Cells
Source: Adv Sci (Weinh). 2023 Apr 7;10(16):2207165. doi: 10.1002/advs.202207165 (PMC10238215; doi:10.1002/advs.202207165)
Supplement: Supplementary file 1 — Supporting Information [file ADVS-10-2207165-s001.pdf]

ADVANCED SCIENCE
Open Access

Supporting Information

for *Adv. Sci.*, DOI 10.1002/advs.202207165

# Molecular/Nanomechanical Insights into Electrostimulation-Inhibited Energy Metabolism Mechanisms and Cytoskeleton Damage of Cancer Cells

*Guohua Qi, Miaomiao Zhang, Jilin Tang* and Yongdong Jin**

# Supporting Information

**Molecular/Nanomechanical Insights into Electrostimulation-Inhibited Energy Metabolism Mechanisms and Cytoskeleton Damage of Cancer Cells**

Guohua Qi,[#] Miaomiao Zhang,[#] Jilin Tang*, Yongdong Jin*

## Experimental section

### Materials and Chemicals

The sodium citrate, silver nitrate, paraformaldehyde, glucose, and 4-Mercaptopyridine (4-MPy) were bought from Sigma-Aldrich. The fluorescein isothiocyanate (FITC) and $[Ru(dpp)_3]Cl_2$ were obtained from Aladdin. Glucose oxidase was bought from Yuan Ye Biotechnology Co., Ltd (Shanghai); The 5, 5′, 6, 6′-Tetrachloro-1, 1′, 3, 3′-tetraethyl-imidacarbocyanine（JC-1）and 4', 6-diamidino-2-phenylindole (DAPI) were bought from the Invitrogen (Carlsbad, CA). The methylthionine chloride, 3-(4, 5-dimethyl-2-thiazolyl)-2, 5-diphenyl-2-H-tetrazolium bromide (MTT) were purchased from Aladdin. The dimethyl sulfoxide (DMSO) was purchased from Key-GenBioTech. The ATP assay kit, pyruvate assay kit, TRITC-phalloidin and DMEM (without glucose and phenol red) were obtained from Solarbio. $NADP^+$/NADPH and $NAD^+$/NADH assay Kits were purchased from Beyotime. 2-NBDG (glucose analog) was obtained from Glpbio. The Dulbecco’s modified Eagle’s medium (DMEM), fetal bovine serum (FBS), 0.25% trypsin/2.2 mM EDTA solution and antibiotic solution contained 200 units $mL^{-1}$ penicillin and 200 units $mL^{-1}$streptomycin were purchased from VivaCell, Shanghai, China. Glucose transporter protein antibody was bought from Abcam. All the solutions were prepared through the ultrapure water which was obtained using a Millipore Milli-Q water purification system (Billerica, MA), with an electric resistance >18.25 MΩ.

### Instruments

The silver nanoparticles (AgNPs) were characterized through Hitachi 600 transmission electron microscope (TEM). The UV-Vis absorption spectra were taken using a Lambda 750 spectrophotometer (Perkin-Elmer). The concentrations of the NPs were detected using the

ICP-OES (Thermo Scientific Icap 6300). The dynamic light scattering (DLS) was performed on an instrument (Zetasizer Nano ZS 90) purchased from British Ma Erwen co., Ltd. The fluorescence emission spectrum was obtained on a Shimadzu RF-5301PC spectrophotometer. The enzyme-labelled meter was purchased from Tecan, which was used to detect the absorbance of formazan for assessing cell cytotoxicity. The electrochemical measurement was performed on the CHI 660C electrochemical workstation. An inverted microscope (Leica DMI6000B, Germany) with an external double channel optical system. The SERS spectra were collected during the 633 nm laser exposure period by a confocal Raman system (LabRAM ARAMIS, HORIBA JobinYvon, USA). The morphology and nanomechanical properties of cells were characterized using the Peak Force QNM mode (Nanoscope v9.3) of BioScope Resolve AFM (Bruker, USA).

**Preparation of AgNPs**

Firstly, the $AgNO_3$ (0.009 g) was dispersed into deionized water (50 mL) and then to heat under stirring. After the mixed solution was boiled, 2 mL of sodium citrate (Wt 1%) was quickly added into the reaction solution to boil continuously for 40 min. Finally, the mixed solution was cooled at room temperature and stored in the dark for further use.

**Glucose sensing**

Typically, the G-nanoprobes were dispersed into different concentration of glucose (0, 4, 8, 12 and 16 mM) for 4 h at 37 $^{0}$C water bath. After that, the mixed solution was centrifugate at 6500 rpm for 10 min. The supernatant was detected for estimating concentration variations of glucose using the fluorescence spectra. Meanwhile, the deposit was redispersed into deionized water for SERS detection.

**Cell culture**

The HeLa (cervical cancer cells), HepG2 (liver cancer cells), MCF-7 (breast cancer cells), H8 (cervical epithelium immortalized cells) and mammary epithelial cells (MCF-10A) were obtained from the American Type Culture Collection (ATCC, USA). The four cell lines

(HeLa, HepG2, MCF-7 and H8 cells) were cultured in the Dulbecco's Modified Eagle's Medium (DMEM) and the MCF-10A cells were cultured in an appropriative medium (Procell, CM-0525) supplemented with 10% fetal bovine serum (FBS), 100 μg/mL streptomycin and 100 U/mL penicillin at 37 °C in a humidified atmosphere containing 5% $CO_2$.

**MTT assay for detecting cytotoxicity of G-nanoprobes**

Firstly, the MCF-7 cells ($1\times10^4$ cells in each well) were planted in a 96-hole plate for 12 h. Subsequently, the cells were washed using the PBS by three times and the different concentrations of G-nanoprobes were added into each well. The cells were cultured continuously for 24 h in sugar-free complete medium. After that, the cells were cleaned through PBS by three times. Then a 10 μL of MTT solution (5 mg/mL) was added into each well and was incubated at 37 °C for another 4 h in the $CO_2$ incubator. Finally, a 150 μL of DMSO after removing the supernatant medium was added into each well after reaction to dissolve purple formazan crystals. The absorbance of the wells was tested on a microplate reader with a measurement wavelength of 570 nm.

**Electrical stimulation for cells**

The ITO electrode was first disinfected with ultraviolet radiation for 30 min to capture the cells because the surfaces of cells are negatively charged. Cells ($1.0 \times 10^5$) on the ITO glass were then cultured for 12 h at 37 °C in a humidified atmosphere containing 5% $CO_2$, which was set as working electrode. The electrical stimulation platform of cells was performed in a standard three-electrode system on a CHI 660E electrochemical workstation at room temperature. The NaCl (0.9 wt %) and complete medium at a volume ratio of 4:1 was selected as electrolyte. A Pt sheet and Ag/AgCl (KCl saturated) were used as the counter electrode and reference electrode, respectively.

**Intracellular ROS production**

2', 7'-dichlorofuorescein (DCFH) converted from 2', 7'-dichlorofluorescin diacetate (DCFH-DA) was employed to evaluate the ROS generation within MCF-7 cells during ES process.

Before the ES, the MCF-7 cells were incubated with DCFH-DA for 20 min and then the cells were washed using PBS by three times. After that, the cells were treated with different voltages (0, 0.4, 0.8, and 1.2 V) for 5 min and continue to cultivate for 30 min. ROS production within cells during different groups were observed and recorded through the fluorescence microscope.

**Testing $O_2$ content variations with MCF-7 cells after ES under different voltages**

Firstly, the cells were seeded on the ITO glass for ES under different voltages and then the cells were washed using the PBS three times. After that, the cells were stained with the $O_2$ probe of RDPP (10 μM) for 4 h at 37 °C in a humidified atmosphere containing 5% $CO_2$. Finally, the cells were cleaned with PBS three times and observed and recorded through the fluorescence microscope at 40× objective.

**SERS detection of intracellular glucose**

Briefly, the MCF-7 cells were seeded on the ITO glass to culture overnight. The cells were washed using the PBS and incubated with G-nanoprobes in sugar-free complete medium for 4 h. After that, the cells were cleaned through PBS by three times and treated with ES under different time (0, 1, 3 and 5 min) and voltages (0, 0.4, 0.8 and 1.2 V). The SERS spectra of single cells were collected during the NIR laser exposure period by a confocal Raman system with a 7.1 mW/633 nm laser. The spot size was 1.5~3 μm and outstretched scan spectra with a spectral from 400 to 1800 $cm^{-1}$ were recorded with an integration time of 10 s and one accumulation.

**Live/dead cell staining**

The cells were seeded on the ITO glass to incubate for 12 h and the cells were washed using PBS by three times. After that, the cells were treated under different voltages (0, 0.4, 0.8 and 1.2 V) for 5 min. The cells electrodes were cleaned using PBS by three times, which stained with mixed solution containing Calcein AM (2 μM) and propidium iodide (PI, 4 μM) for 20

min. After that, the cells were cleaned three times using the PBS and recorded using Leica DMI6000B microscope with a fluorescence detector with 10× objective.

**Detection of mitochondrial membrane potential (MMP)**

Typically, the MCF-7 cells were treated by ES under different voltages above mentioned. Then, the cells were cleaned using the PBS by three times and stained with commercial dye of JC-1 assay kit for 20 min. After that, the cells were washed using the PBS by three times and the fluorescence imaging of cells were observed and collected using Leica DMI6000B microscope with a fluorescence detector with 20× objective (EM: 510-540 nm (JC-1 monomer) and EM: 570-620 nm (JC-1 aggregate)).

**Detection of ATP within MCF-7 cells during ES**

The commercialized ATP kit assay was used to check the energy changes within MCF-7 cells during ES process. First, the MCF-7 cells were treated with different voltages (0, 0.4, 0.8 and 1.2 V) for 5 min. After that, the cells were washed using the PBS by three times. The trypsase solution was applied for the preparation of the cell suspension from cells covered on the ITO glass. After that, the cells were collected and washed using the PBS by three times. The number of cells after the stimulation under different times and voltages was calculated using the cell count. The cells were collected into centrifuge tubes to remove the supernatant. Then, 200 μL of cell extract solution were added into tubes for ultrasonication in an ice-bath for 1 min. After that, the mixed solution was centrifugated at 6000 rpm in 4 $^{0}$C for 10 min and the supernatant was added into another tube. After that, 50 μL of chloroform was added into tubes and adequately shocked to mix well. Subsequently, the mixed solution was centrifugated at 6000 rpm in 4 $^{0}$C for 3 min and the supernatant was kept to further detection in 4 $^{0}$C. The final supernatant was mixed with work solution according to instruction to detect the absorption at 340 nm using enzyme-labelled meter.

**Intracellular pyruvate detection**

Typically, the cells were collected in tubes after the ES under different conditions. The cells were washed and centrifugated at 1000 rpm for 5 min. The supernatant was removed after centrifugation and added extraction solution for ultrasonication at ice-bath. After that, the solution was placed for 30 min and then was centrifugated at 8000 ×g for 10 min. The supernatant was retained for detection. The pyruvate detection within supernatant was performed following the instruction procedure.

**GLUT1 expression of MCF-7 cells after ES**

The glucose transporter protein expression of the cells was conducted using the immunofluorescence. Briefly, the MCF-7 cells were washed using PBS by three times after ES under different groups. The control groups were not treated with ES. The cells were fixed with 4% paraformaldehyde for 20 min. After that, the cells were cleaned using the PBS by three times. To enhance cell permeability, the cells were incubated with triton-X 100 for 20 min at room temperature and then washed three times using PBS again. The cells were exposed to a blocking buffer (1% BSA in PBS) for 1 h at room temperature. Subsequently, the cells were incubated with glucose transporter protein antibodies labeled with Alexa Fluor® 647 (dilution 2:1000) overnight at 4 ºC. Next day, the cells nuclei were stained with DAPI for 10 min. Finally, the cells were rinsed using PBS for three times and then imaged by the fluorescence microscope.

**Labeling and analysis of cytoskeletal states**

TRITC-phalloidin can specifically bind to F-actin of eukaryotes that could be used to easily visualize the states of cytoskeletal. To label the intracellular F-actin, after removing the complete cell culture medium, the electrically stimulated cells were washed three times with PBS and incubated with 1.5 mL of 4% paraformaldehyde solution for 20 min. Then, the cells were permeabilized with 0.5% triton-X 100 for 30 min and washed three times with PBS. After that, 1 mL of 0.1 mM TRITC-phalloidin was added to label cells for 20 min at room temperature. Then, 1 mL of 5 ng/mL Hoechst 33342 was used to mark the cell nucleus. After

washing three times with PBS, each sample was mounted with anti-fade mounting medium and fluorescence imaging was performed using a 100 x oil lens of a confocal fluorescence microscope.

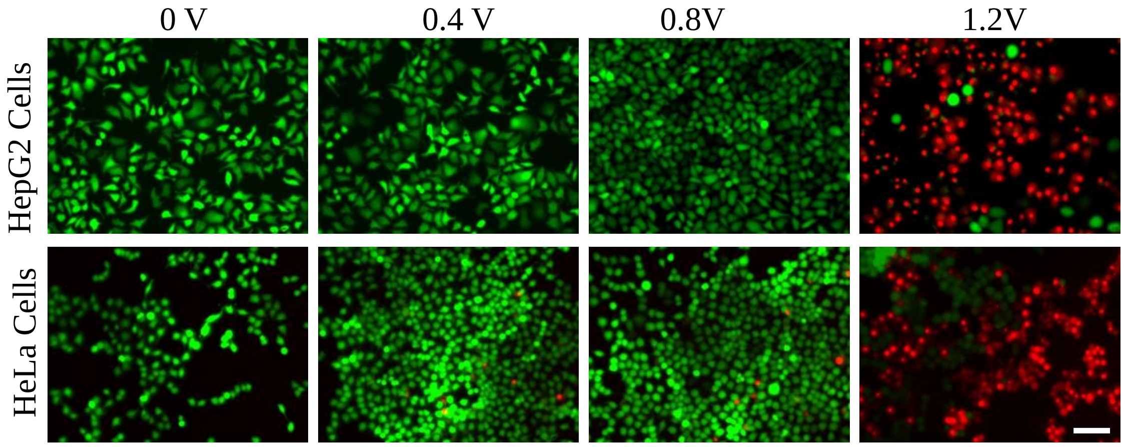


**Figure S1.** Fluorescence images of HepG2 and HeLa cells stained with living/dead cell staining dye after the ES treatment for 5 min under different voltages. The scale bar is 50 μm.

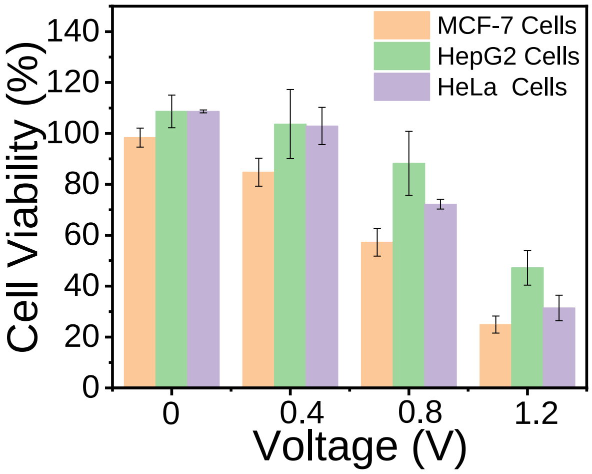


**Figure S2.** The cell viability of three tested cell lines (MCF-7, HepG2 and HeLa cells) after ES treatmet with different voltages for 5 min, tested by MTT assay.

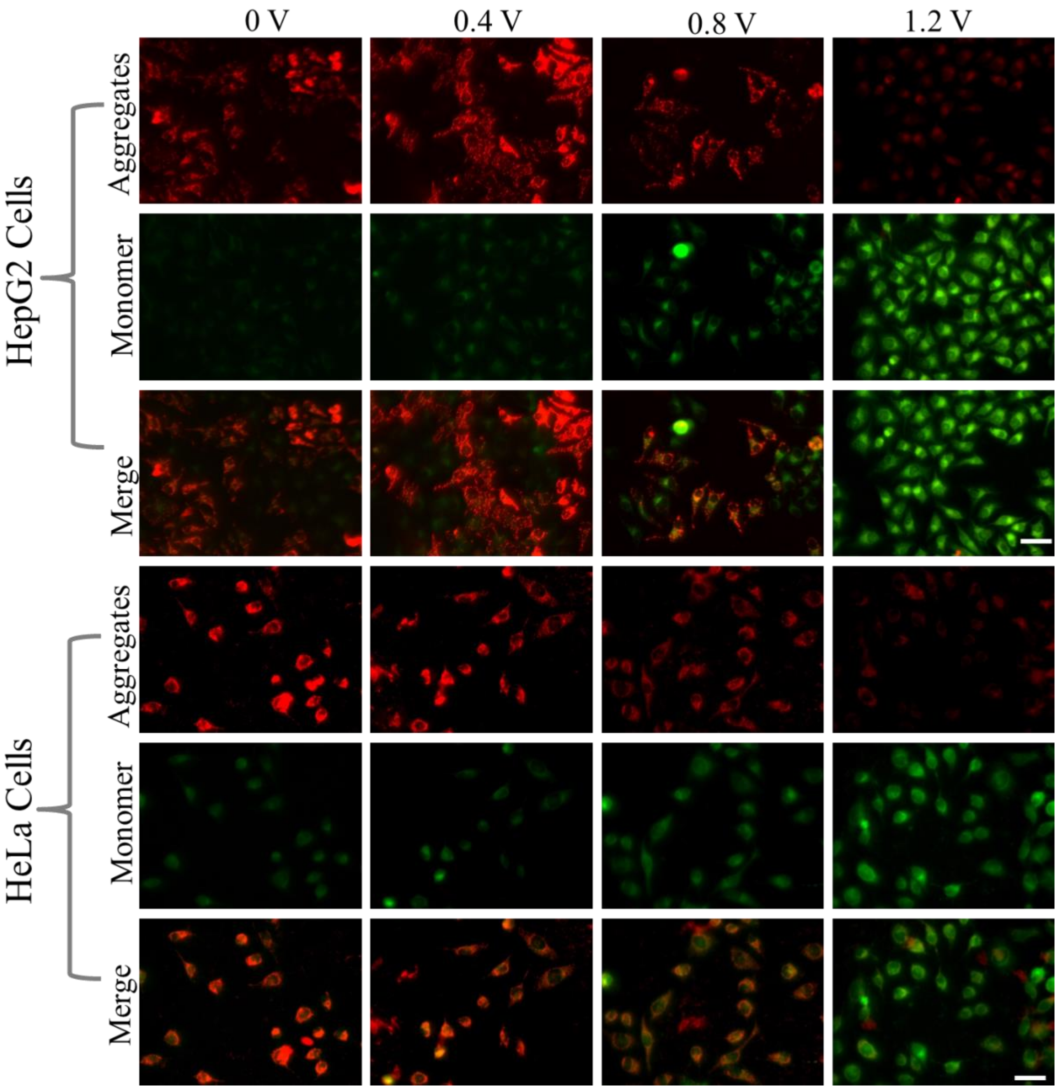


**Figure S3.** Fluorescence imaging of MMP within HepG2 and HeLa cells using JC-1 assay kit after the ES treatment with different voltages for 5 min. The scale bar is 50 μm.

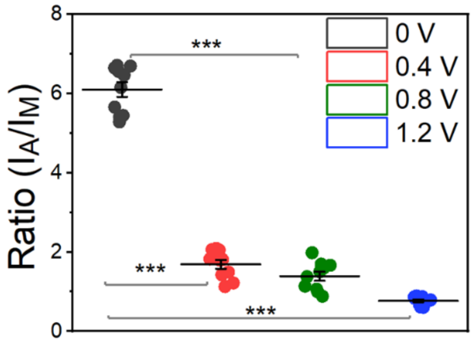


**Figure S4.** The ratio of fluorescence intensity between the monomer (λem = 537 nm) and aggregated (λem = 587 nm) JC-1 within MCF-7 cells after the ES under different voltage for 5 min. *** $P<0.001$.

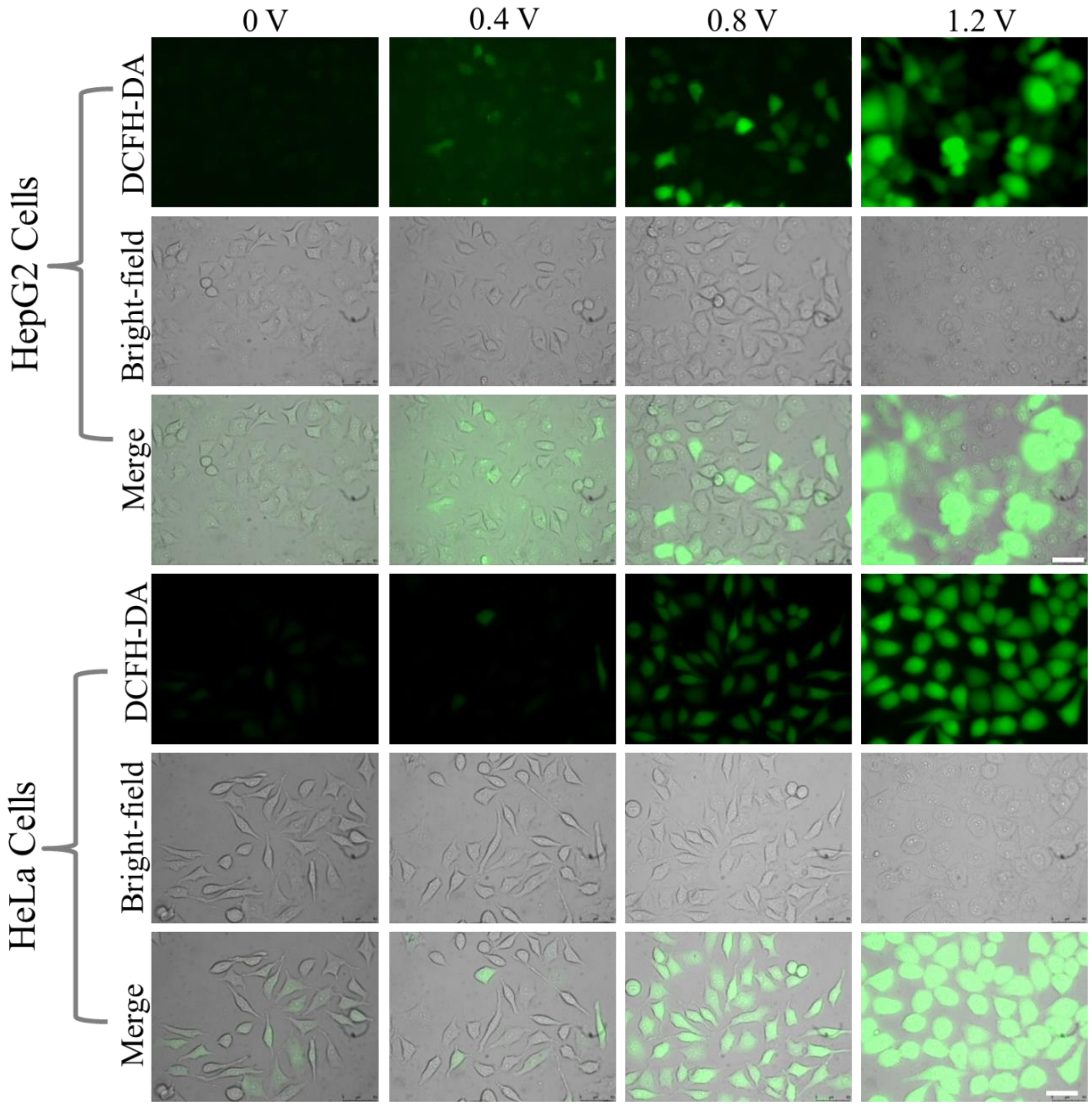


**Figure S5.** The ROS fluorescence imaging of HepG2 and HeLa cells stained with DCFH-DA after the ES under different voltages for 5 min. The scale bar is 50 μm.

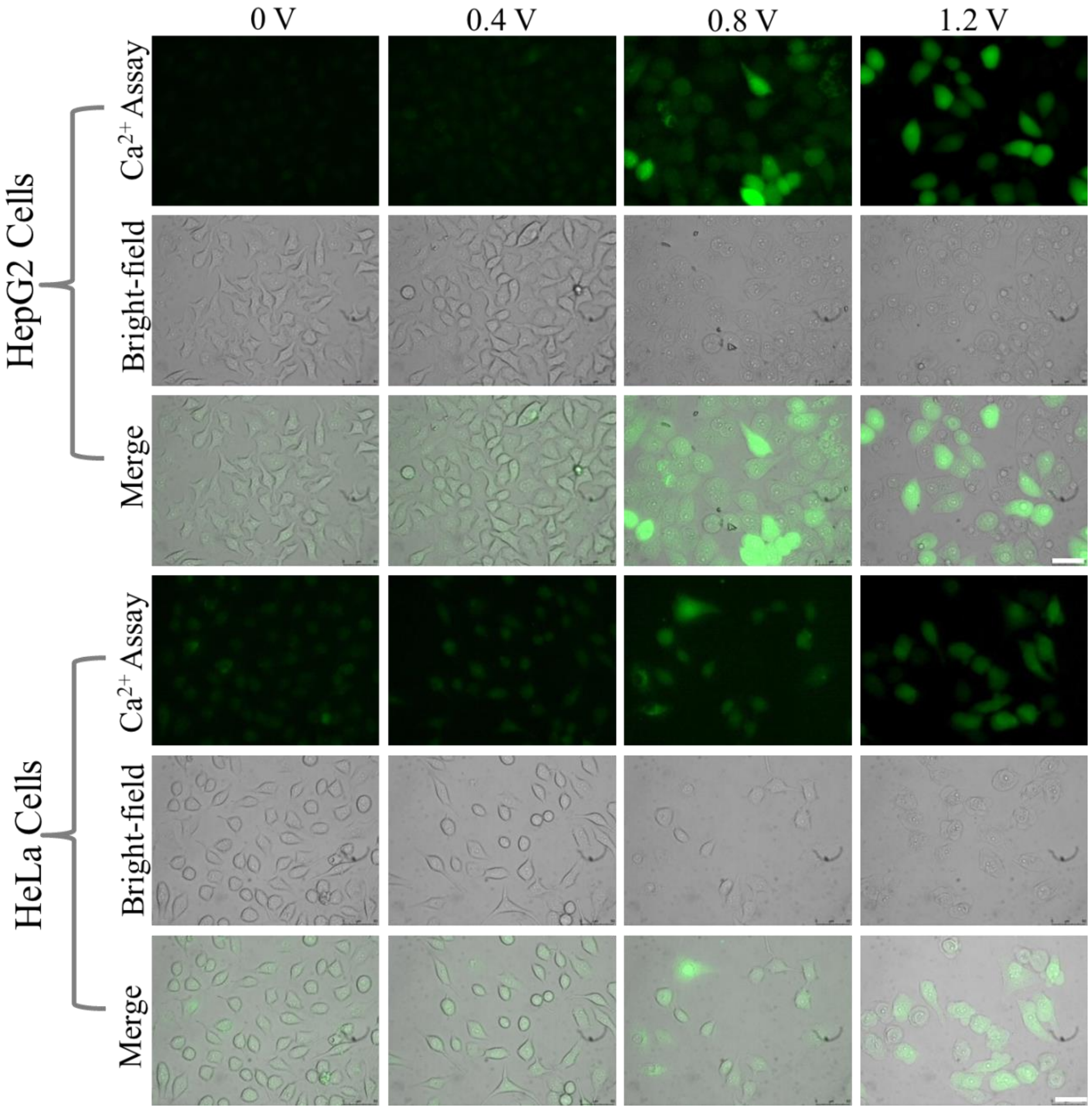


**Figure S6.** The $Ca^{2+}$ fluorescence imaging within HepG2 and HeLa cells stained with $Ca^{2+}$ assay kit after the ES for 5 min under different voltages. The scale bar is 50 μm.

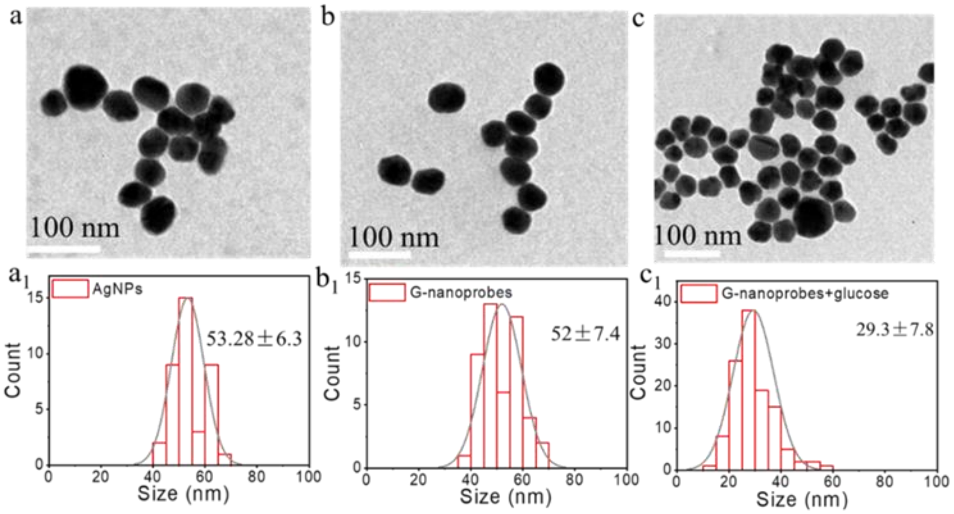


**Figure S7.** (a)-(c) TEM images of AgNPs (a), and G-nanoprobes before (b) and after (c) the reaction with glucose (10 mM) for 2 h. (a1)-(c1) The corresponding size distributions of AgNPS, and G-nanoprobes before and after the reaction with glucose.

To detect the glucose within MCF-7 cells after ES treatment at different voltages, the glucose nanoprobes (G-nanoprobes) were prepared through fluorescein isothiocyanate (FITC) that also selected as Raman reporter and glucose oxidase co-modified on the surface of AgNPs. Typically, the morphology of AgNPs, G-nanoprobes before and after reaction with glucose was checked using transmission electron microscope (TEM) and their size distributions were calculated (Figure S7). The results indicated that the morphology and size of AgNPs basically remained unchanged before and after the modification, while the mean size of G-nanoprobes was significantly decreased from 52 ±7.4 nm to 29.3±7.8 nm after reaction with glucose, because of following reactions:

$$Glucose + O_2 + H_2O \rightarrow gluconic\ acid + H_2O_2 \quad (1);$$

$$2Ag + H_2O_2 \rightarrow 2Ag^+ + 2OH^- \quad (2);$$

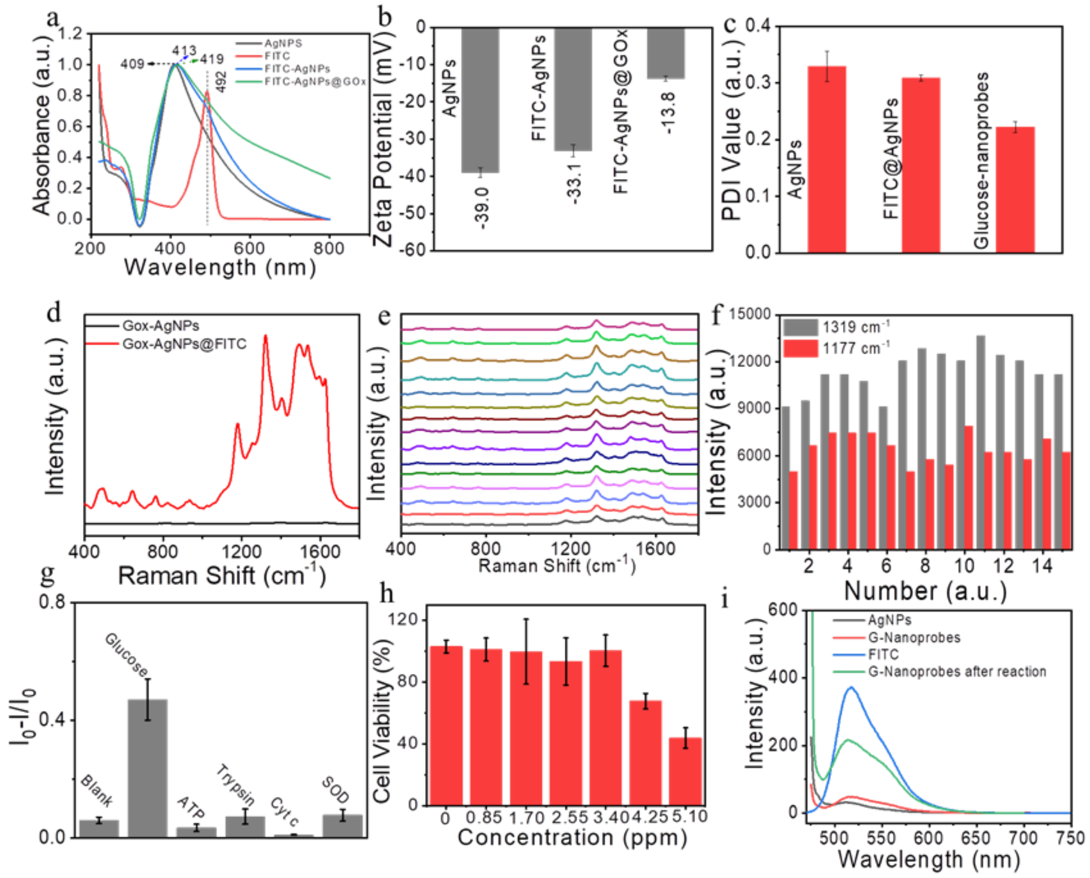


**Figure S8.** (a) The UV-Vis spectra and (b) zeta potential of AgNPs after the different surface modifications. (c) The PDI values of AgNPs, FITC-AgNPs and G-nanoprobes. (d) SERS spectra of GOx-AgNPs and G-nanoprobes. (e) SERS spectra of G-nanoprobes detected for 15 points selected randomly. (f) SERS intensity at 1177 and 1319 $cm^{-1}$ for 15 points selected randomly. (g) SERS intensity at 1323 $cm^{-1}$ in response to other substances for selectivity test. (h) Cell viability of MCF-7 cells treated with G-nanoprobes with different concentrations. (i) Fluorescence spectra of AgNPs, FITC, G-nanoprobes, and solution of g-nanoprobes reacted with 4 mM glucose.

To prove that the G-nanoprobes were successfully prepared, the UV-Vis spectra of AgNPs were performed under different modification process (Figure S8a). The results showed that the local plasmon absorption peak of AgNPs emerged obviously red shift from 413 to 419 nm. Meanwhile, the zeta potential of AgNPs was gradually reduced during the modification process (Figure S8b), which further confirmed the successful preparation of the G-nanoprobes.

The polydispersity index (PDI) values were performed by dynamic light scattering (DLS) analysis of the nanoprobes which are less than 0.4 (Figure S8c), suitable for biological system studies.[1] The palpable SERS signal from FITC was collected (Figure S8d). Moreover, the G-nanoprobes provided good repetitiveness (Figure S8e&f), with relative standard deviation (RSD) values at 1323 and 1177 $cm^{-1}$ of 11.8% and 14.5%, less than 20%.[2] Moreover, the superior selectivity and biocompatibility of G-nanoprobes at 3.4 ppm were investigated (Figure S8g and 8h). The sensitive response of G-nanoprobes to glucose was verified from fluorescence spectra (Figure S8i). All these results fully proved the successful preparation of the designer G-nanoprobe.

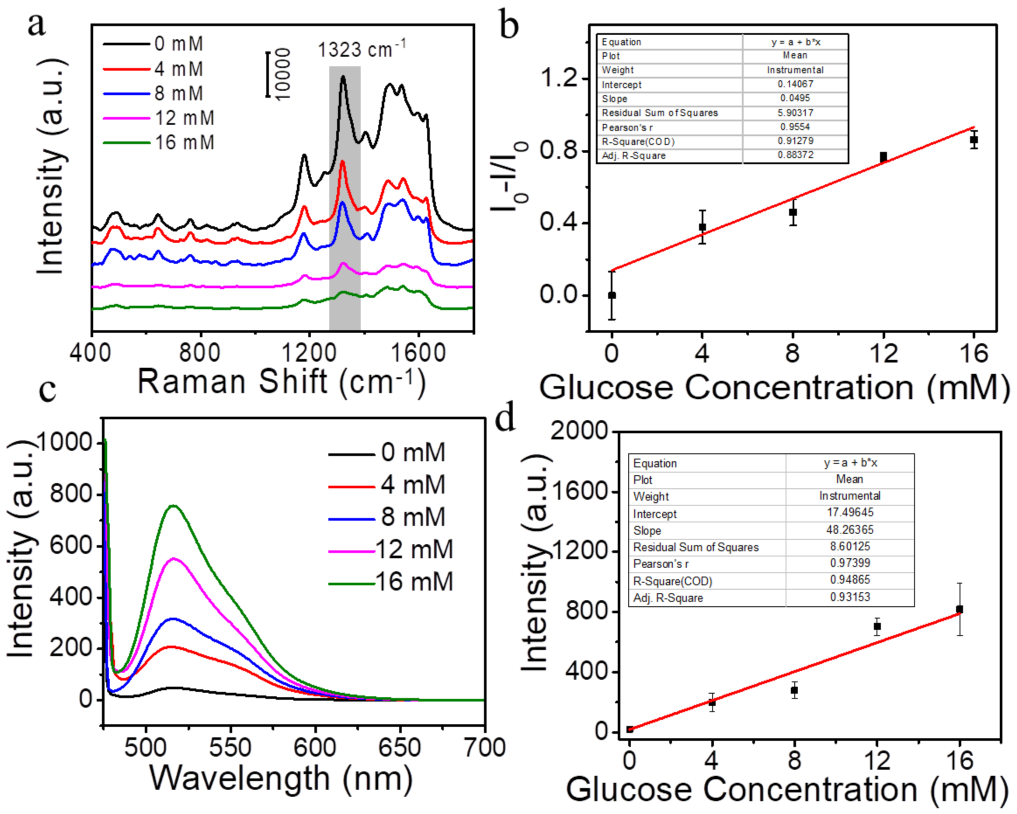


**Figure S9.** (a) and (c) The SERS and fluorescence spectra of G-nanoprobes after the incubation with different concentrations of glucose (0, 4, 8, 12, and 16 mM). (b) and (d) Linear relationships between SERS intensity at 1323 $cm^{-1}$ or fluorescence intensity at 516 nm and glucose concentrations.

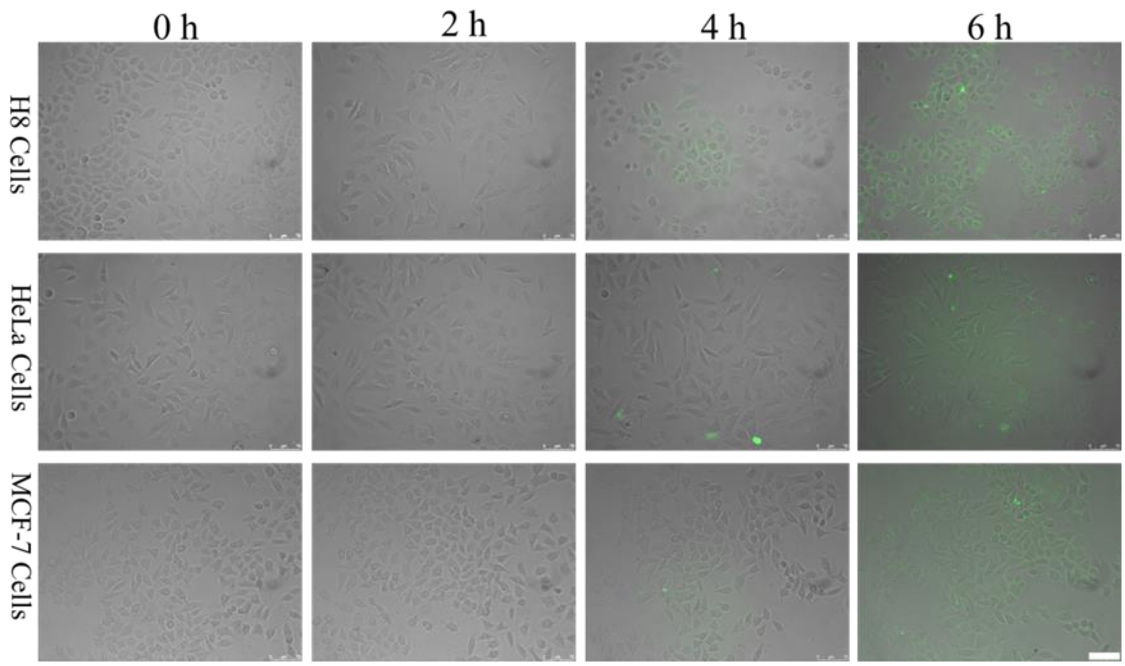


**Figure S10.** Fluorescence imaging of G-nanoprobes after entering into three tested cell lines (H8, HeLa and MCF-7 cells) under different time. The scale bar is 75 μm.

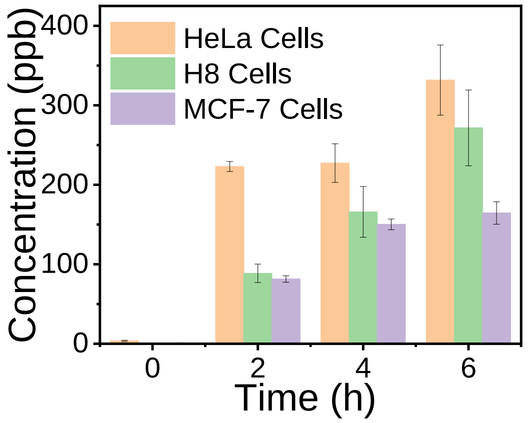


**Figure S11**. The contents of Ag within three tested cell lines after the G-nanoprobes co-cultured with glucose-free DMEM for different time detected using ICP-MS.

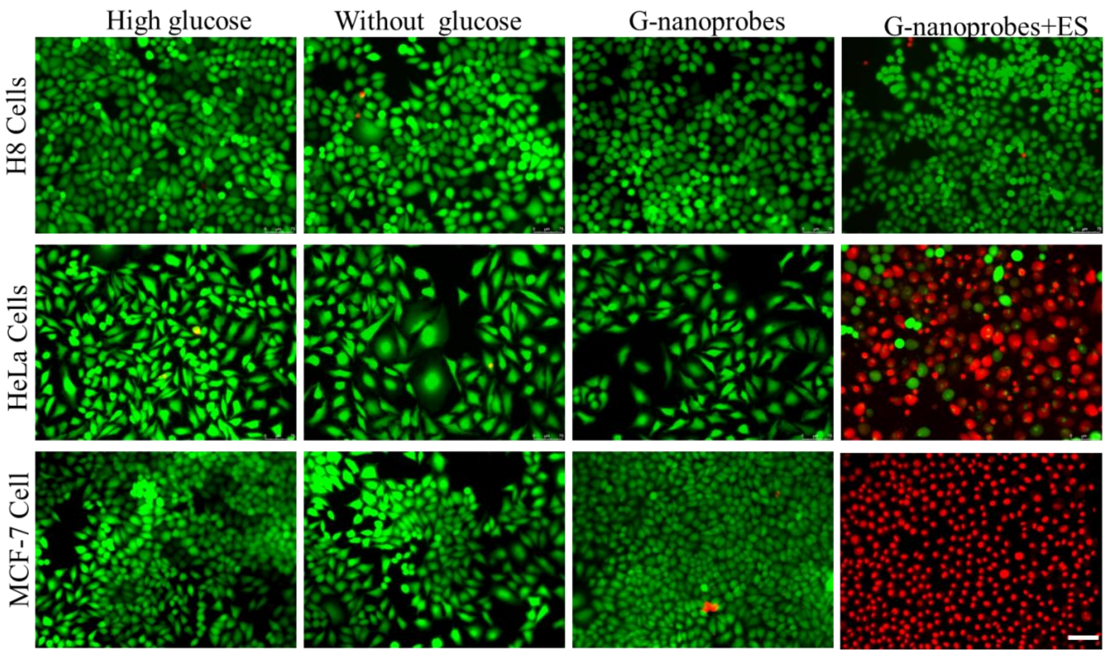


**Figure S12.** The fluorescence imaging of three tested cell lines (H8, HeLa and MCF-7 cells) stained with AM/PI assay kit after the incubation with DMEM in the presence or absence of high concentration glucose, glucose-free DMEM and incubated with G-nanoprobes dissolved with DMEM without glucose before and after the ES at 1.2 V for 5 min. The scale bar is 50 μm.

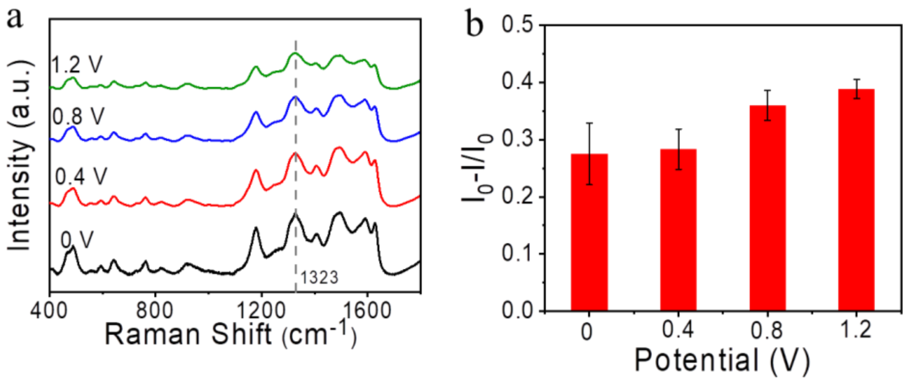


**Figure S13.** The SERS spectra and SERS intensity at 1323 $cm^{-1}$ of G-nanoprobes within MCF-7 cells after the ES under different voltage (0, 0.4, 0.8 and 1.2 V) for 5 min.

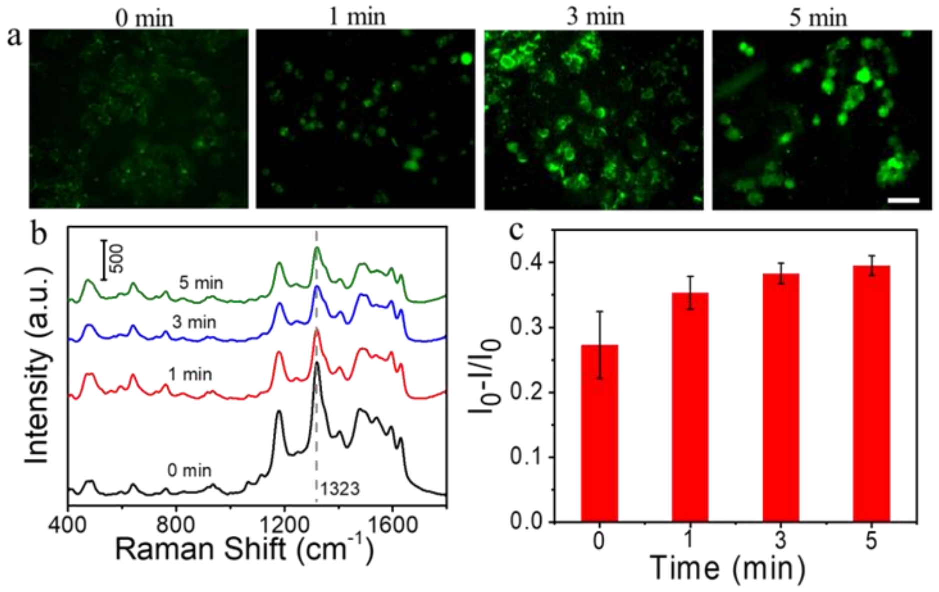


**Figure S14.** Fluorescence imaging of intracellular glucose within MCF-7 cells treated with 1.2 V under different time (0, 1, 3 and 5 min). (b)-(c) The SERS spectra and SERS intensity at 1323 $cm^{-1}$ of G-nanoprobes within MCF-7 cells treated with 1.2 V under different time.

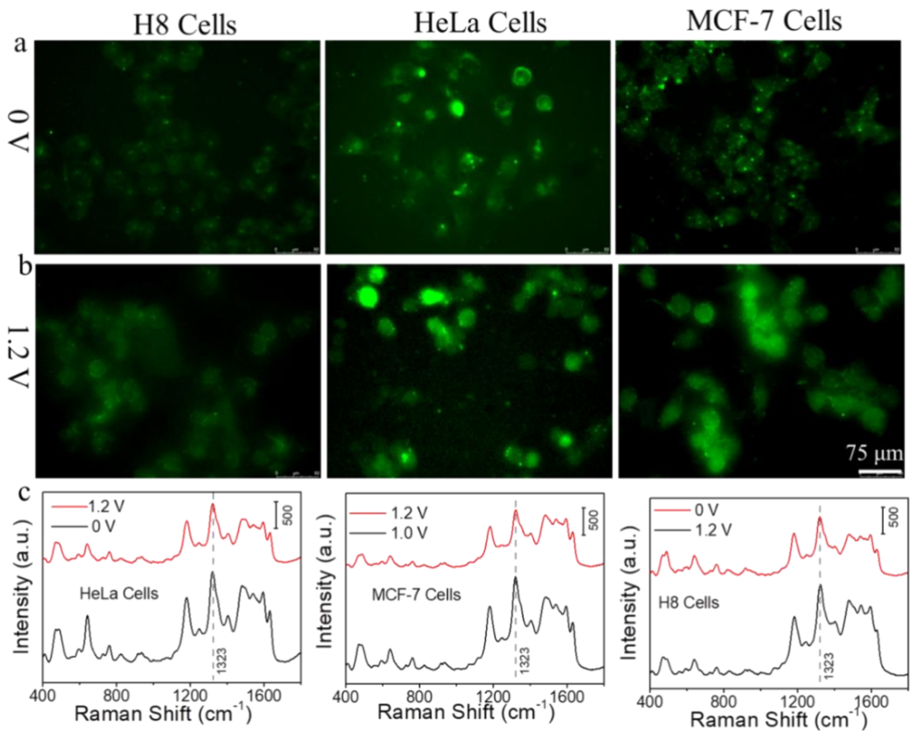


**Figure S15.** (a-b)The fluorescence imaging of intracellular glucose within three cell lines (H8, HeLa and MCF-7 cells) tested before and after the ES at 1.2 V for 5 min. The scale bar is 75 μm. (c) SERS spectra of G-nanoprobes incubated with three cell lines (H8, HeLa and MCF-7 cells) before and after the ES at 1.2 V for 5 min.

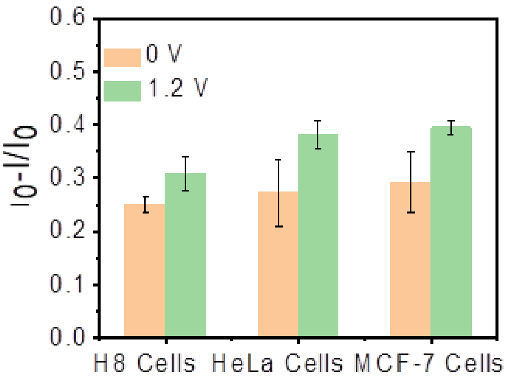


**Figure S16.** The content variation of glucose with three tested cell lines (MCF-7, HeLa and H8 cells) before and after the ES under 1.2 V for 5 min.

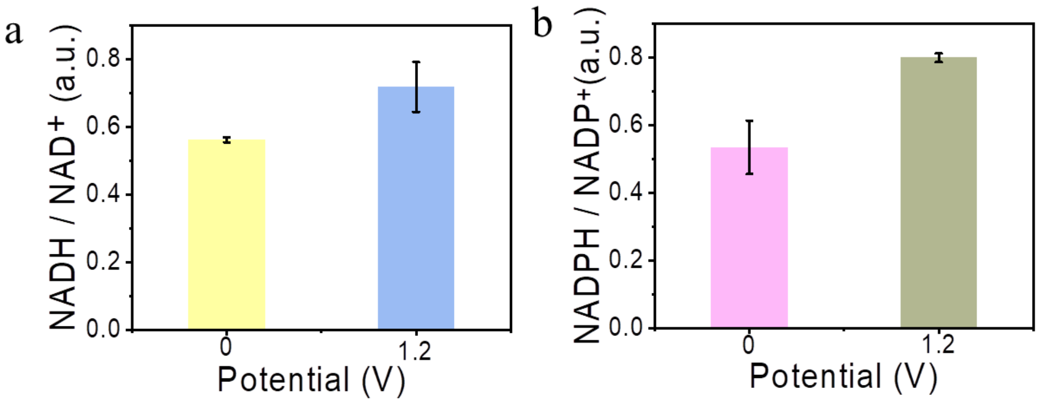


**Figure S17.** The ratios of NADH/ $NAD^+$ and NADPH/ $NADP^+$ calculated within MCF-7 cells before and after the ES under 1.2 V for 5 min.

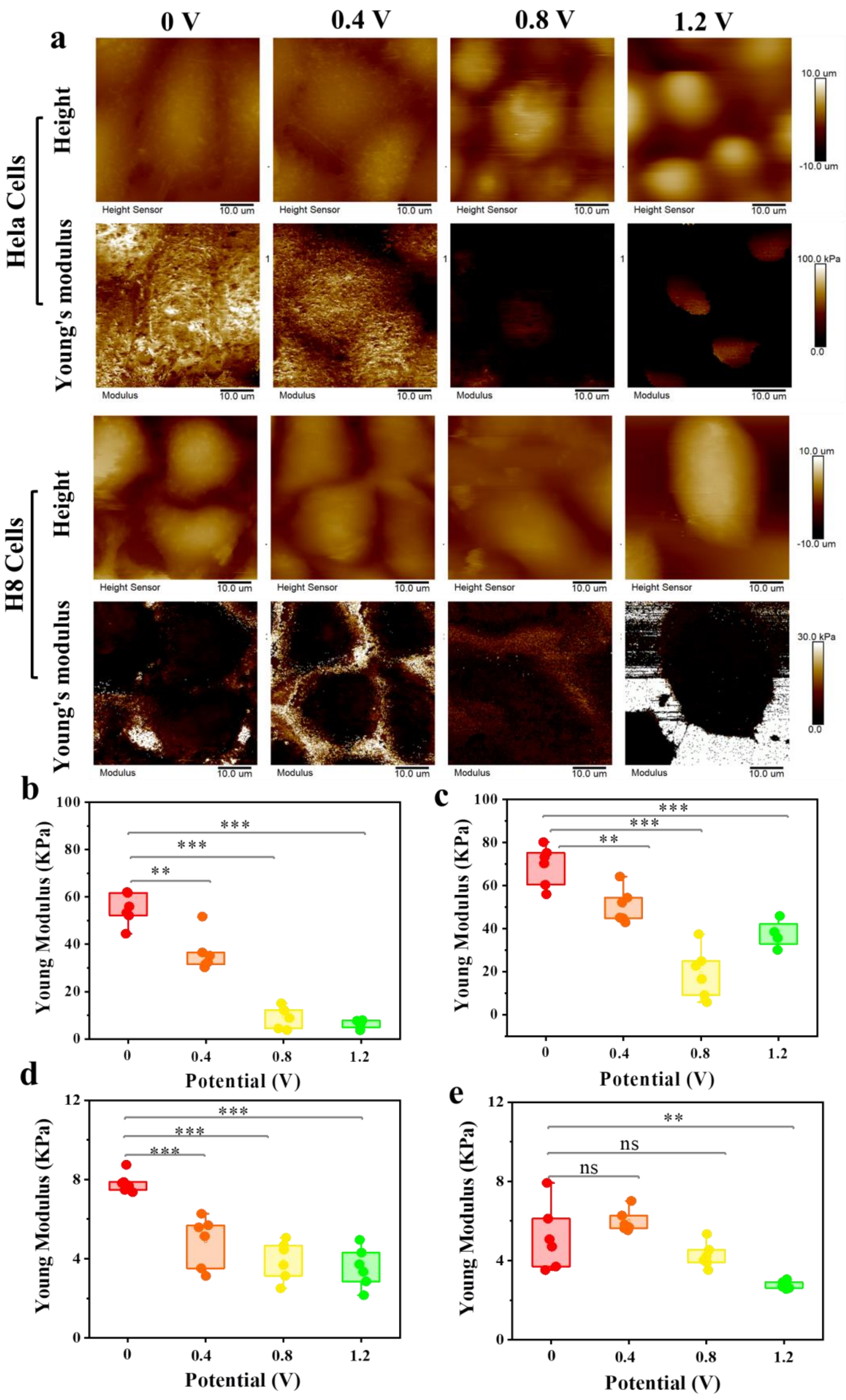


**Figure S18**. (a) Representative AFM topography images and corresponding Young's modulus images of HeLa and H8 cells. (b)-(c)The statistical analysis of perinuclear and central nuclear areas Young's modulus from HeLa cells treated with different voltages (0, 0.4, 0.8 and 1.2 V) for 5 min. (d)-(e) The statistical analysis of perinuclear and central nuclear areas Young's modulus from H8 cells treated with different voltages for 5 min.Values are expressed as means ± SD. **P<0.005 and ***P<0.0001 represents the statistical significance.

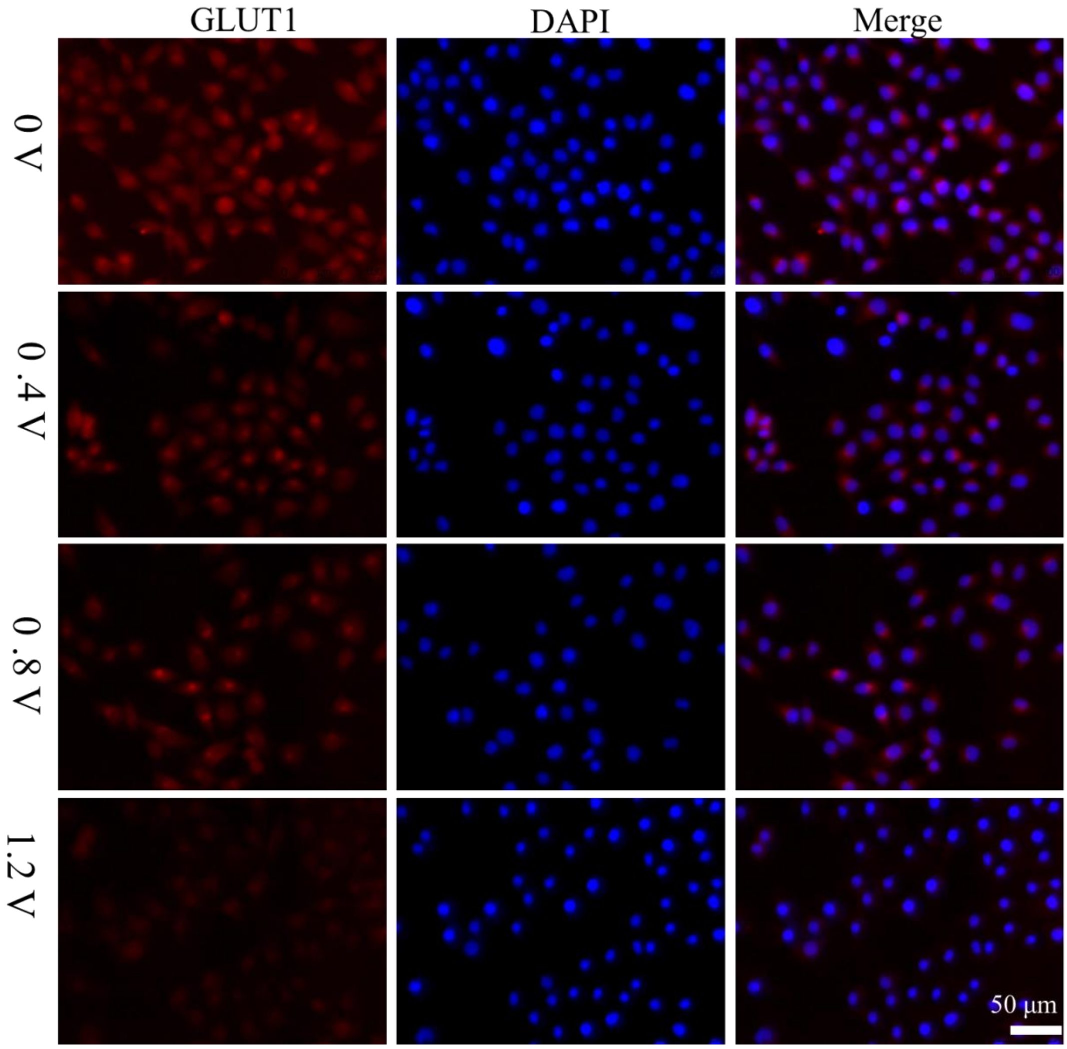


**Figure S19.** Immunofluorescence imaging of GLUT1 from MCF-7 cells after the ES treatment with different voltages for 5 min. The cell nucleus was stained using the DAPI. The scale bar is 50 μm.

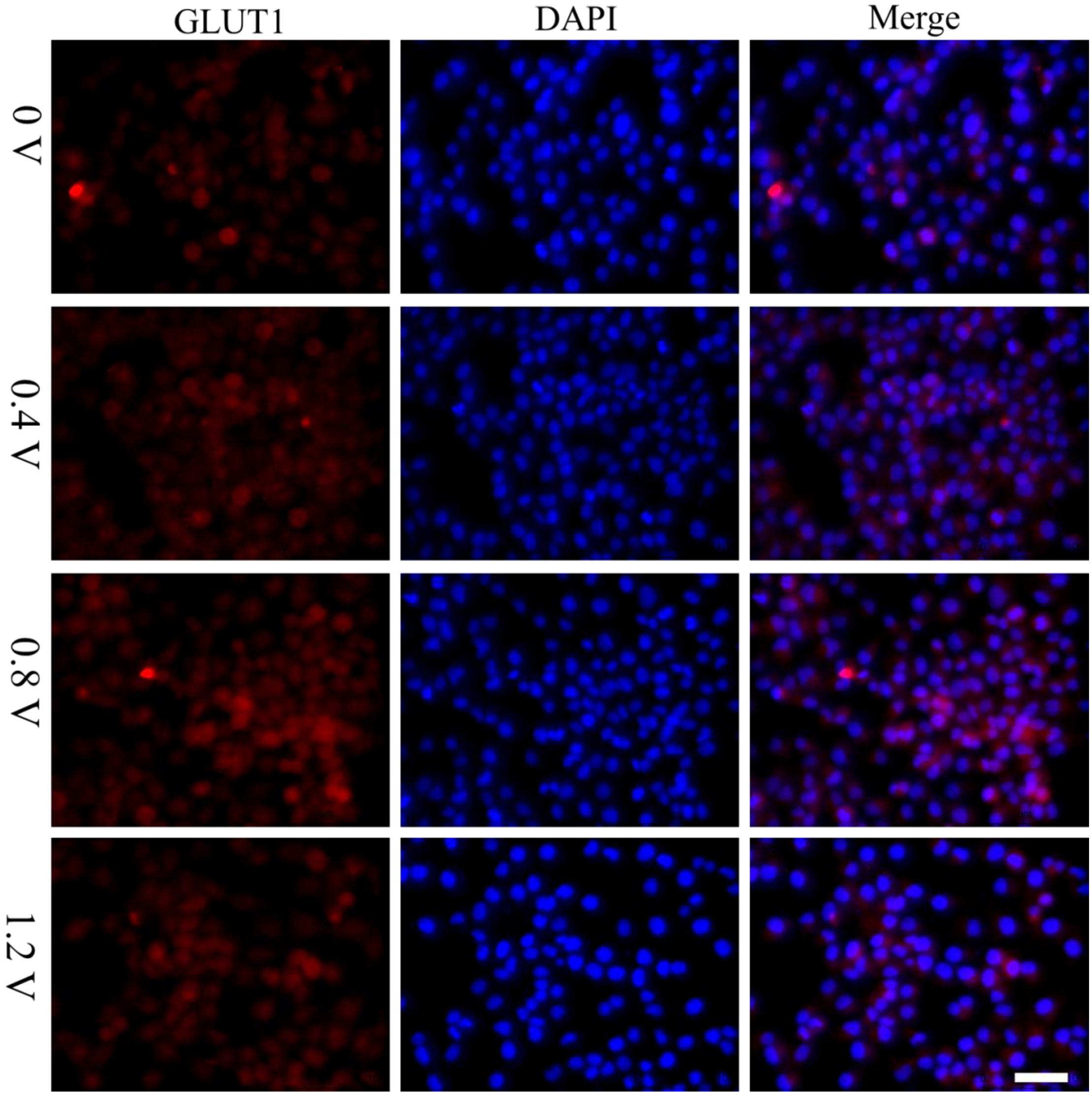


**Figure S20.** Immunofluorescence imaging of GLUT1 from H8 cells after the ES treatment with different voltages for 5 min. The cell nucleus was stained using the DAPI. The scale bar is 50 μm.

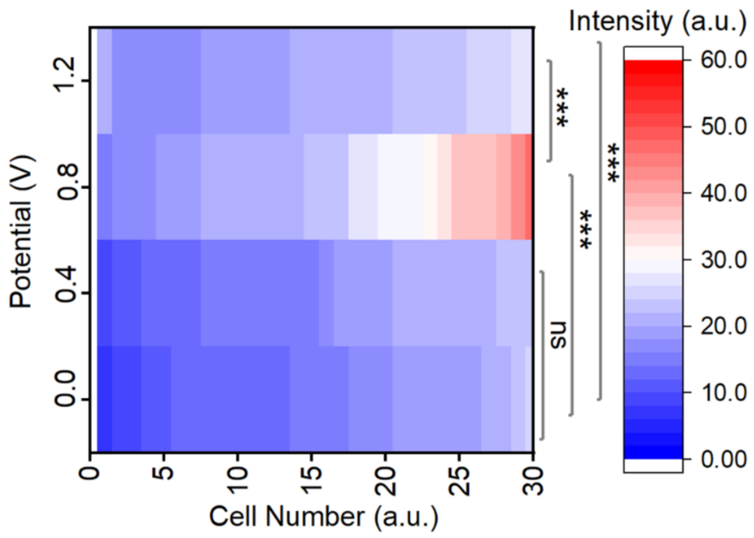


**Figure S21.** The FL intensity heat maps of GLUT1 on cell membrane of H8 cells treated with ES under different voltages for 5 min.

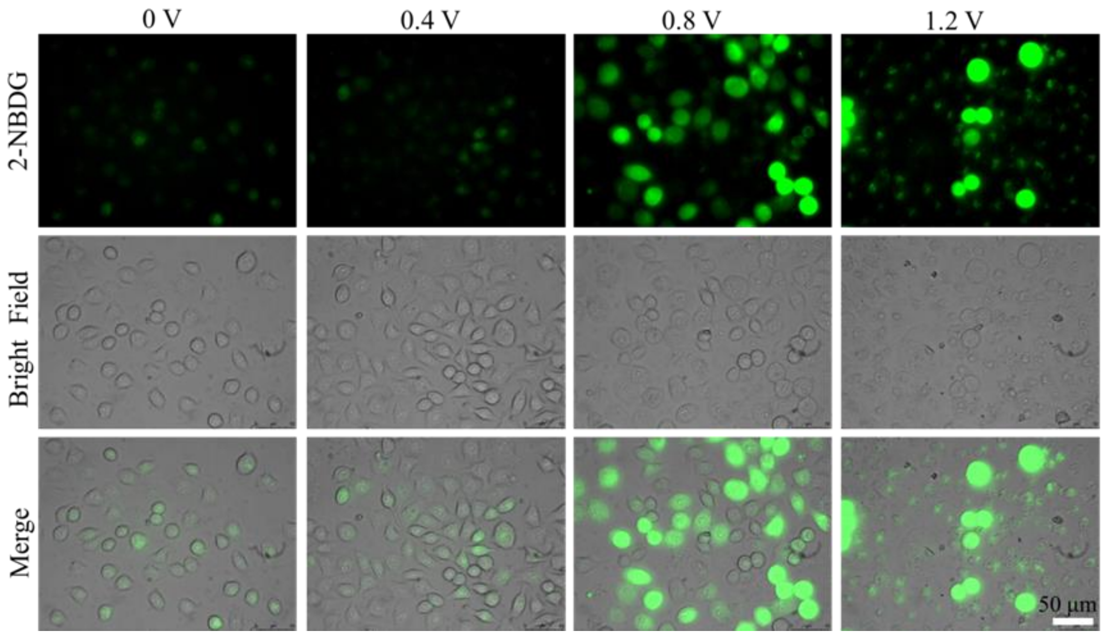


**Figure S22.** The fluorescence imaging of MCF-7 cells stained with 2-NBDG for estimating the glucose uptake ability of cells after the ES under different voltages for 5 min.

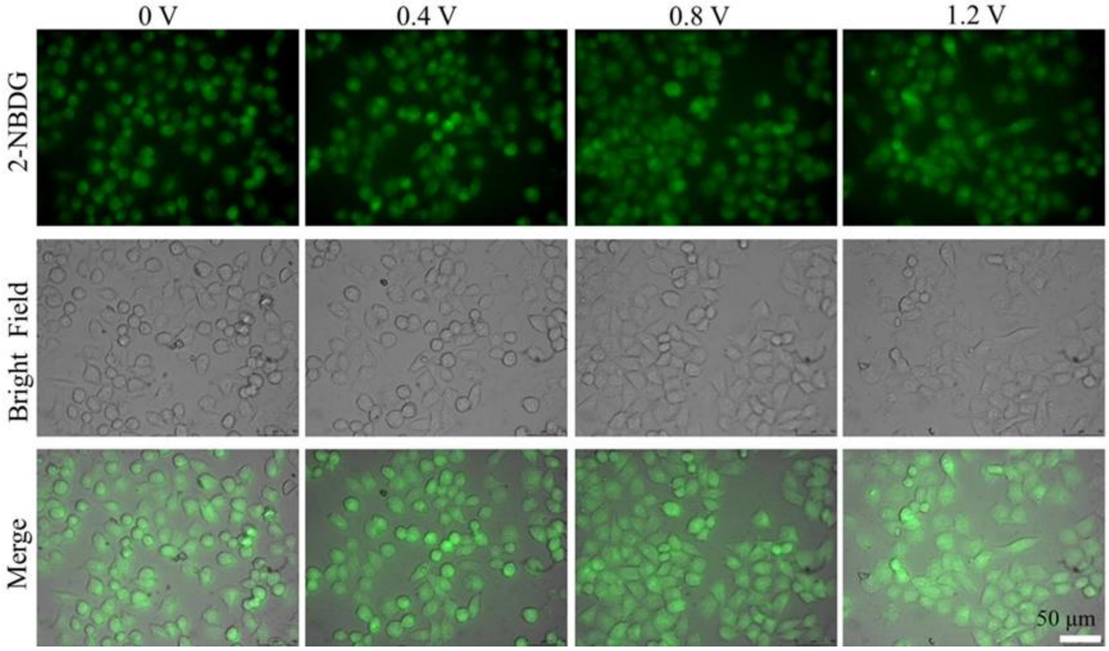


**Figure S23.** The fluorescence imaging of H8 cells stained with 2-NBDG for estimating the ability of the cells to uptake glucose after the ES under different voltages for 5 min.

**References:**

[1] G. H. Qi, D. Sun, Y. Tai, C. Xu, Y. Zhang, D. D. Wang, K. S. Ma, S. P. Xu, Y. D. Jin, *Anal. Chem.***2020**, *92*, 7861.

[2] G. H. Qi, H. J. Li, Y. Zhang, C. P. Li, S. P. Xu, M. M. Wang, Y. D. Jin, *Anal. Chem.* **2019**, *91*, 1408.
